# The rehabilitation of face recognition impairments: a critical review and future directions

**DOI:** 10.3389/fnhum.2014.00491

**Published:** 2014-07-23

**Authors:** Sarah Bate, Rachel J. Bennetts

**Affiliations:** Department of Psychology, Faculty of Science and Technology, Bournemouth UniversityPoole, UK

**Keywords:** face recognition, prosopagnosia, neurorehabilitation, cognitive training, face processing

## Abstract

While much research has investigated the neural and cognitive characteristics of face recognition impairments (prosopagnosia), much less work has examined their rehabilitation. In this paper, we present a critical analysis of the studies that have attempted to improve face-processing skills in acquired and developmental prosopagnosia, and place them in the context of the wider neurorehabilitation literature. First, we examine whether neuroplasticity within the typical face-processing system varies across the lifespan, in order to examine whether timing of intervention may be crucial. Second, we examine reports of interventions in acquired prosopagnosia, where training in compensatory strategies has had some success. Third, we examine reports of interventions in developmental prosopagnosia, where compensatory training in children and remedial training in adults have both been successful. However, the gains are somewhat limited—compensatory strategies have resulted in labored recognition techniques and limited generalization to untrained faces, and remedial techniques require longer periods of training and result in limited maintenance of gains. Critically, intervention suitability and outcome in both forms of the condition likely depends on a complex interaction of factors, including prosopagnosia severity, the precise functional locus of the impairment, and individual differences such as age. Finally, we discuss future directions in the rehabilitation of prosopagnosia, and the possibility of boosting the effects of cognitive training programmes by simultaneous administration of oxytocin or non-invasive brain stimulation. We conclude that future work using more systematic methods and larger participant groups is clearly required, and in the case of developmental prosopagnosia, there is an urgent need to develop early detection and remediation tools for children, in order to optimize intervention outcome.

## Introduction

Prosopagnosia is a cognitive condition characterized by a relatively selective deficit in face recognition. Traditionally the disorder has been described in a small number of individuals who acquire face recognition difficulties following neurological injury or illness, typically affecting occipitotemporal regions (De Renzi et al., 1994; Gainotti and Marra, 2011). Although acquired prosopagnosia (AP) in its purest form is a rare condition (Gloning et al., 1967; Zihl and von Cramon, 1986), many more individuals with brain damage are believed to experience moderate-to-severe face-processing deficits alongside other cognitive impairments (Hécaen and Angelergues, 1962; Valentine et al., 2006). Further, as many as 2.9% (Bowles et al., 2009) of the population may experience developmental prosopagnosia (DP)—an apparently parallel form of the disorder that occurs in the absence of neurological injury or lower-level visual deficits (e.g., Duchaine and Nakayama, 2005; Bate and Cook, 2012). While some people cope relatively well with prosopagnosia, it can have a devastating effect on an individual's everyday social and occupational functioning (Yardley et al., 2008). Hence, exploration of the remediation of prosopagnosia is an urgent clinical issue that, unfortunately, has received little attention to date. It is important to note that rehabilitation is not necessary in all cases of prosopagnosia—some people with DP cope relatively well, and many devise their own strategies to recognize the people around them (e.g., Fine, 2012). However, Yardley et al. (2008) note that the majority of their participants reported negative psychosocial experiences related to DP, particularly at a younger age. As such, investigations into the effectiveness of remediation techniques—especially those used in children—are important both on a theoretical and a practical level.

The few studies that have attempted to remedy face-processing deficits in individuals with AP or DP are summarized in Table 1. In the current paper, we present a critical review of substantive published attempts to rehabilitate AP and DP, examining both the design of each training programme and the research participants themselves, in an attempt to place the findings in the context of the wider neurorehabilitation literature. It has been argued that the main aim of neuropsychological rehabilitation is to reduce the impact of impairments on everyday living, whether through restoration of function or the adoption of coping strategies (Wilson, 2003). In the context of face recognition, rehabilitation may therefore encourage an individual to develop compensatory strategies that aid person recognition, or attempt to restore—or, in the case of DP, to develop—normal face-processing mechanisms via more extensive visuo-cognitive training (referred to as “remedial training” in this paper). Although the neurorehabilitation literature is vast, it has seldom been applied to disorders of face-processing. As such, current research offers little guidance as to which approach (compensatory or remedial) may be more effective in prosopagnosia, or the factors that may influence the effectiveness of each method. Therefore, the main aim of this review is to provide guidance on this issue.

**Table 1:** **A summary of published research reports that have examined intervention techniques in acquired or developmental prosopagnosia**.

**Reference**	**Incident age (yrs)**	**Intervention age (yrs)**	**Lesion**	**Functional implication**	**Intervention technique**	**Success**	**Generalized gains (lab)**	**Gains to everyday life**	**Gains maintained**
**ACQUIRED**									
Ellis and Young, 1988	3	8	Implied RH lesion and bilateral occipital damage	Perceptual	Remedial	No	–	–	–
De Haan et al., 1991b	19	23	Bilateral temporo-occipital junction	Perceptual and mnemonic	Compensatory	No	–	–	–
Polster and Rapcsak, 1996	61	68	Right temporo-occipital	Perceptual	Compensatory	Yes	No	Unknown	Unknown
Francis et al., 2002	19	21	Right temporal	Semantic and mnemonic	Compensatory	Yes	Yes	No	Unknown
Powell et al., 2008	<51	>51	Bilateral occipital, left temporal, and frontal	Perceptual	Compensatory	Yes	Unknown	Unknown	Unknown
**DEVELOPMENTAL**									
Brunsdon et al., 2006	–	8	–	Perceptual	Compensatory	Yes	No	Yes	Yes
DeGutis et al., 2007	–	48	–	Perceptual	Remedial	Yes	Yes	Yes	No
Schmalzl et al., 2008	–	4	–	Perceptual	Compensatory	Yes	Unknown	Yes	Yes
DeGutis et al., 2014	–	*M* = 35, *N* = 24	–	Various	Remedial	Yes	Yes	Yes	Unknown

First, we address the question of whether the typical face-processing system retains neuroplasticity throughout the lifespan – in other words, is there evidence that the face-processing system might be able to learn or improve face-specific processing mechanisms at any point in time, or should prosopagnosia interventions focus primarily on critical periods of development or the development of compensatory strategies? Second, we examine intervention studies in AP and DP, with a specific focus on factors that may affect success, including the nature of the disorder, the type of intervention, and individual differences between participants. Finally, we discuss future directions in the rehabilitation of prosopagnosia.

## Does the typical face-processing system remain plastic throughout the lifespan?

The term “neuroplasticity” typically refers to a neural system's capacity to learn new skills or improve existing capabilities, either during normal development or after neurological damage (e.g., Huttenlocher, 2002). Traditionally, there have been two main theories on neuroplasticity (Thomas, 2003). The first proposes that an innate blueprint specializes cognitive systems for a particular function, which emerges during critical periods within development. This perspective suggests that once the relevant neural structures have been specialized for their purpose, any damage can only be overcome by the adoption of compensatory behavioral strategies. In face-processing, this might take the form of recognizing people based on individual facial features, or using additional semantic cues during face encoding. In contrast, the other viewpoint proposes that the brain retains plasticity throughout the lifespan, and hidden reserves may aid the acquisition of new skills or compensate for damage—providing that appropriate intervention techniques are used. Drawing on the available neurorehabilitation literature, Thomas (2003) concludes that the brain's structures are not irreversibly determined by an innate plan, but plasticity is nevertheless limited. Further, these limits may fluctuate throughout development, and are not necessarily consistent across different neural systems. Therefore, before examining neuroplasticity in the context of prosopagnosia, it follows that neuroplasticity within the typical face-processing system should be examined. That is, is it theoretically possibly that face recognition skills can be improved at any point in the lifespan, or does research using neurotypical participants indicate that any plasticity in the neural face-processing system is short-lived following birth?

A dominant theory of the development of face-processing posits that crude brain circuits become specialized for face recognition in response to early visual experience with faces (the “perceptual narrowing” hypothesis: Nelson, 2001). Evidence supporting this theory comes from findings that very young infants can discriminate between monkey and other-race faces, whereas older infants and adults no longer have this ability (e.g., Pascalis et al., 2002; Kelly et al., 2007). Although these findings suggest some plasticity in the face-processing system in the first few months of life, Nelson suggests that early specialization of neural tissue for face-processing may lead to a lack of plasticity in later years.

Behavioral studies tracking the development of face recognition skills also suggest that specialized face processing systems emerge early in life. In a review of developmental studies conducted to date, Crookes and McKone (2009) conclude that adult-like face-processing strategies are obtained by early childhood in qualitative if not quantitative terms, suggesting a window for plasticity only within the first years of life. For example, one key marker of mature face-processing skills is the ability to process faces on a holistic basis, taking into account the overall configuration of facial features and the spacing between them (Maurer et al., 2002). As Crookes and McKone note, evidence of holistic processing has been observed in children as young as 3 or 4 years using classical paradigms such as the face inversion effect (Sangrigoli and de Schonen, 2004), the composite effect (de Heering et al., 2007; Macchi Cassia et al., 2009a), the part-whole effect for upright but not inverted faces (Pellicano and Rhodes, 2003), and tests that assess sensitivity to spacing between facial features (McKone and Boyer, 2006; Pellicano et al., 2006). A second marker of adult-like face-processing skills is the “inner-feature advantage” whereby adults are more proficient at recognizing familiar faces from the inner compared to the outer features (Ellis et al., 1979; Young et al., 1985)—a preference that has also been observed in children as young as 5 years of age (Wilson et al., 2007). Further, Pozzulo and Lindsay (1998) reported a meta-analysis that summarized findings from eye-witness studies that used children as participants. In agreement with the above studies, the authors noted that children as young as 5 years of age display adult-like performance in their ability to identify perpetrators from target-present (but not target-absent) line-ups. These studies therefore indicate that, despite evidence indicating a large increase in face recognition ability throughout childhood (presumably due to the need for more generalized mechanisms to develop), there is no qualitative change in face perception beyond 4–5 years of age. In fact, given increasing evidence that even infants are capable of holistic processing (Cohen and Cashon, 2001; Bhatt et al., 2005; Hayden et al., 2007) it is possible that face-processing skills are fully-developed at a very early age, implying a limit on plasticity beyond early childhood. This idea is supported by studies of adolescents and adults who were born with dense cataracts—despite the fact that the cataracts were removed before 7 months of age, participants show abnormal face-processing skills (Le Grand et al., 2001, 2004) but normal object discrimination (Robbins et al., 2010), indicating that early visual input is particularly important for the development of face-processing mechanisms.

While early visual input may be necessary for the initial development of face-processing mechanisms, it remains possible that these mechanisms can be refined or altered later in life. Despite evidence of early commitment to face-specific regions, neuroimaging studies suggest that the cortical face-processing system (Haxby et al., 2000; Gobbini and Haxby, 2007) continues to develop well into adolescence. For instance, Passarotti et al. (2003) found more diverse activation in the fusiform region for children as opposed to adults. Similarly, Gathers et al. (2004) reported that activation in the fusiform gyrus is not greater for faces compared with objects until 10 years of age, although they did note such activation more posteriorly in the inferior occipital region. Other studies suggest that both activation of the core face-processing system and connectivity between the different neural areas changes between the ages of 7 and 11 years (Cohen Kadosh et al., 2011, 2013). Event-related potential (ERP) components also continue to mature through late childhood into early adolescence: Taylor et al. (2004) reported that face inversion did not influence the face-specific N170 response until 8–11 years of age. While these findings raise the possibility that plasticity may remain in the face-processing system at least until adolescence, De Schonen et al. (2005) warn that plasticity during typical brain development is most likely due to modification of synaptic organization, rather than redistribution of face-processing mechanisms to other cortical regions. Hence, these findings do not imply that other neural areas can simply take over face-processing following brain damage.

There are also several lines of evidence that support the idea that the face-processing system may retain some plasticity even in adulthood. For instance, Germine et al. (2010) tested over 60,000 participants aged from pre-adolescence to middle-age on their ability to learn new faces. In three experiments, Germine and colleagues found that face learning ability improves up until the age of 30, although the recognition of inverted faces and name recognition peak at a much earlier age. Other evidence supporting plasticity in the adult face-processing system comes from studies of the other-race effect, or the finding that we are better at recognizing faces from our own race than those from other races (e.g., Malpass and Kravitz, 1969). Critically, one of the explanations for this effect is based on the presumption that the phenomenon reflects the lack of experience the viewer has had with faces from the other race (Meissner and Brigham, 2001; Hancock and Rhodes, 2008). Although the effect has been observed in infants as young as 3 months of age (e.g., Sangrigoli and de Schonen, 2004; Kelly et al., 2005, 2007), evidence suggests it remains plastic and reversible even in adulthood. Specifically, Hancock and Rhodes (2008) found a reduced other-race effect, accompanied by increased holistic processing, for participants who reported higher levels of contact with another race (see also Meissner and Brigham, 2001; Sangrigoli et al., 2005; de Heering and Rossion, 2008; Kuefner et al., 2008; Macchi Cassia et al., 2009b; Rhodes et al., 2009, for similar studies of the “own-age bias”). More interestingly, though, training can improve recognition of other-race faces. Tanaka and Pierce (2009) trained Caucasian students to discriminate between African-American and Hispanic faces, and reported an improvement in the recognition of novel stimuli of the same race, along with changes to the N250 ERP component to the other-race faces (see also Elliott et al., 1973; McKone et al., 2007). Notably, McKone et al. (2007) showed normal levels of holistic processing for trained cross-race faces, indicating that training can have an effect on the manner in which faces are processed, not just the accuracy with which they are identified.

In sum, behavioral and neural investigations using typical participants suggest that the face-processing system may retain some plasticity throughout childhood and into adulthood. This raises the possibility that it may be possible to rehabilitate face recognition deficits, at least in some circumstances.

## Neurorehabilitation of acquired prosopagnosia

Anderson et al. (2001) outline two potential means of recovery following brain injury: the spontaneous healing of damaged tissue may lead to reactivation of pre-existing neural pathways, or anatomical reorganization may allow different neural areas to take over the behavioral function of the damaged area. Given evidence that the face-processing system retains some plasticity in adulthood, remediation of face-processing skills following neurological injury may be possible. However, as with any other acquired deficit, it is likely that a number of general constraints will influence the success of intervention. These might include the age at which the lesion was acquired, the severity of the lesion, and the precise functional implications of the lesion. These factors may dictate the type of intervention that is suitable for the individual, and whether it should focus on compensatory rather than remedial training.

### Timing of injury

There is a general view that the developing brain has greater plasticity than the adult brain: Huttenlocher (2002) concludes that, across the neurorehabilitation literature, neuroplasticity in adults has generally been found to be lower than in children. Further, in early development there are higher levels of some genes and proteins that are required for neuronal growth, synaptogenesis and the proliferation of dendritic spines, and these levels significantly reduce with aging (Huttenlocher and Dabholkar, 1997). It therefore follows that compensatory reorganization and transfer of function is more likely after early brain injury (e.g., Elbert et al., 2001).

If plasticity in the developing face-processing system is greater in childhood than in adulthood, one would predict that spontaneous recovery might occur in children to a greater extent than in adults. There have been some instances of recovery of prosopagnosia in adults in the absence of any formal attempts at rehabilitation (e.g., Malone et al., 1982; Lang et al., 2006), but this is by no means consistent: many other cases have found no evidence of improvement or recovery over time (e.g., Sparr et al., 1991; Ogden, 1993; Spillmann et al., 2000). However, work examining the effects of peri- or prenatal injuries on the development of face recognition skills suggests that the infant system may be more plastic following damage than the adult system. For instance, Mancini et al. (1994) found that perinatal unilateral lesions only had mild effects on later face-processing abilities in children ranging in age from 5 to 14 years. In fact, less than half of the children were impaired at face- or object-processing, and face-processing deficits were no more common than object-processing deficits following a right hemisphere lesion.

Although these studies suggest some level of neural reorganization is possible following early damage (see also Ballantyne and Trauner, 1999), it is important to note that age of injury does not have a straightforward relationship with plasticity in the face-processing system. De Schonen et al. (2005) reported a similar study with a group of 5- to 17-year-olds who acquired unilateral posterior lesions involving the temporal cortex during the pre-, peri- or postnatal period. In general, deficits in low-level configural processing were related to face-processing deficits in patients with a lesion acquired before or at birth, when visual experience starts. These findings converge with other work in the neurorehabilitation literature indicating that there may be a U-shaped effect of damage, with prenatal injury leading to the poorest outcome (i.e., with no evidence of transfer of function from the damaged site to intact tissue: Anderson et al., 2001); greater plasticity in early childhood leading to cortical reorganization and greater sparing of function; and more limited plasticity in late adolescence and adulthood. In a similar vein, advanced age at the time of injury may result in less complete recovery compared to younger persons with comparable injuries (Katz and Alexander, 1994). However, the mechanisms of this phenomenon are not known, and it may simply be that increasing age leads to a reduced capacity for compensation or reduced cognitive reserve (Lye and Shores, 2000)—in other words, a more general cognitive decline due to ageing may make it more difficult to relearn old skills or acquire new compensatory strategies.

Another factor that should be taken into account when considering age of injury is the extent of the lesion. Pediatric research has indicated that children with generalized cerebral insult can exhibit both slower recovery and poorer outcome than do adults who suffer similar insults, possibly because attention, memory and learning skills have not been fully developed (Hessen et al., 2007). Without these capacities, the child does not have the tools to efficiently acquire new abilities and cannot progress along the normal pathway of cognitive development.

In sum, evidence from lesion studies suggests that early neurological damage may be more amenable to rehabilitation, but this is modulated by complex interactions with the exact timing and extent of the damage. Currently it is difficult to relate this directly to the prosopagnosia rehabilitation literature, as there is only one study that has attempted to remedy AP in childhood. Ellis and Young (1988) studied an 8-year-old child (KD) who acquired prosopagnosia after anesthetic complications damaged the lateral third and fourth ventricles at 3 years of age (see Table 1). The authors suggest that a persistent left-sided motor weakness implied a right hemisphere lesion, whereas initial loss of vision following the incident suggested bilateral occipital damage. She also had object agnosia, and the underlying deficit seemed to be an inability to construct adequate representations of visual stimuli. The researchers designed a remedial training programme that required KD to complete four tasks over a period of 18 months, including (1) simultaneous matching of photographs of familiar and unfamiliar faces, (2) paired discriminations of computer-generated schematic faces, (3) paired discriminations of digitized images of real faces and (4) the learning of face-name associations. Unfortunately, none of the programmes brought about an improvement in KD's face-processing skills. It is unclear why this programme failed to work, although it is likely that the extensive bilateral damage may have prevented any gains (see section Lesion Size and Location). Notably, this is the only study to date that has attempted to remedy AP acquired as a child, *and* the only study to attempt rehabilitation of a child with AP. As such, it is difficult to assess whether the lack of improvements following this intervention relate to the timing of the injury (3 years of age) or the timing of the intervention (8 years of age), or to comment on the cognitive characteristics/skills that may impact the success of the intervention (e.g., co-occurring object agnosia).

While age of injury may be an important determinant of the success of rehabilitation in AP, the timing of the intervention relative to the injury could also be an important consideration when planning interventions. For example, evidence from the stroke literature suggests that the speed of intervention following the cerebral incident may be fundamental for success. Some studies propose that there are parallels between plasticity mechanisms in the developing nervous system and those occurring in the adult brain immediately following stroke, but that this plasticity diminishes quickly (Biernaskie et al., 2004; Carmichael et al., 2005; Brown et al., 2009). This indicates that the brain may be most receptive to interventions immediately after a stroke, and suggests that early intervention could be crucial in these cases. However, it is currently unknown whether this temporarily increased plasticity extends to (a) the face-processing system, and (b) prosopagnosia acquired from insults other than stroke; it is also unclear whether it interacts with the age of the patient or other factors such as lesion location or severity.

### Lesion size and location

Many causes of the lesions that bring about AP have been reported, including stroke, carbon monoxide poisoning, temporal lobectomy, encephalitis, neoplasm, and head trauma. Further, recent reports have described cases of AP alongside degenerative conditions such as frontotemporal lobar degeneration (Josephs, 2007) and posterior cortical atrophy (McMonangle et al., 2006; Sugimoto et al., 2012), and after temporal lobe atrophy (Joubert et al., 2003; Chan et al., 2009). With such a wide range of preceding causes, attempts to rehabilitate AP must take into account the extent and location of neurological damage, and in particular how different patterns of damage may be associated with different deficits. For example, some recent detailed analyses indicate that the primary site of damage in most cases is to posterior regions of the brain (e.g., Arnott et al., 2008). However, damage to more anterior regions has been reported to bring about “prosopamnesia,” a condition in which patients retain the ability to recognize faces that they knew before the neurological accident, but cannot create stable representations of new faces in memory (e.g., Crane and Milner, 2002). As no attempts have been made to rehabilitate prosopamnesia, it is unknown whether one type of impairment is more amenable to intervention.

Lateralization of the lesion is another potentially important consideration. It was traditionally thought that AP results from unilateral damage to the right hemisphere, particularly the right occipitotemporal area. In line with this hypothesis, De Renzi et al. (1994) reported unilateral occipitotemporal lesions in three cases of AP, and cited 27 previously reported cases that presented with similar damage. However, some reports suggest the disorder can also result from unilateral left hemisphere lesions (Mattson et al., 2000; Barton, 2008), although De Renzi et al. (1987) suggested that prosopagnosia resulting from left hemisphere lesions can result in a more variable pattern of symptoms, and Gainotti and Marra (2011) suggest that AP cases involving left and right hemisphere lesions present with different patterns of functional impairment. This suggests that right and left hemisphere cases may warrant different methods of intervention (see section Identifying the Functional Impairment).

AP has also been reported in the context of bilateral damage (e.g., Damasio et al., 1982; Barton et al., 2002; Boutsen and Humphreys, 2002). Some authors have suggested that unilateral lesions bring about more selective impairments in face-processing, whereas bilateral lesions cause more extensive disruption (Warrington and James, 1967; Boeri and Salmaggi, 1994). This latter suggestion seems logical, given that, when only one hemisphere is affected, it is plausible that neural areas in the undamaged hemisphere might compensate for lost abilities at least to some degree; whereas no such compensation can occur in individuals with damage to both sides of the brain. Indeed, in the more general neurorehabilitation literature, functional plasticity is generally not observed in cases of bilateral damage, and greater damage tends to lead to worse outcomes. Broadly speaking, plasticity is most associated with focal lesions where true recovery with relatively little compensation is possible, presumably because some of the tissue that is crucial for function is unaffected by the lesion (Moon et al., 2009). While large focal lesions may also be associated with good recovery, this tends to only occur when damage is unilateral.

When looking at instances of spontaneous recovery from AP, there is some indication that this occurred following unilateral (Glowic and Violon, 1981; Lang et al., 2006) rather than bilateral (Sparr et al., 1991; Ogden, 1993) damage. When it comes to formal interventions (summarized in Table 1) two of the three AP studies that have reported some success involve patients with unilateral damage (i.e., Polster and Rapcsak, 1996; Francis et al., 2002); the other study reporting improvement involved a patient with bilateral damage that did not consistently affect the same areas of the brain (Powell et al., 2008). The two interventions that failed to show improvement (Ellis and Young, 1988; De Haan et al., 1991b) both involved patients with apparently more extensive bilateral damage.

### Identifying the functional impairment

**Figure 1:**
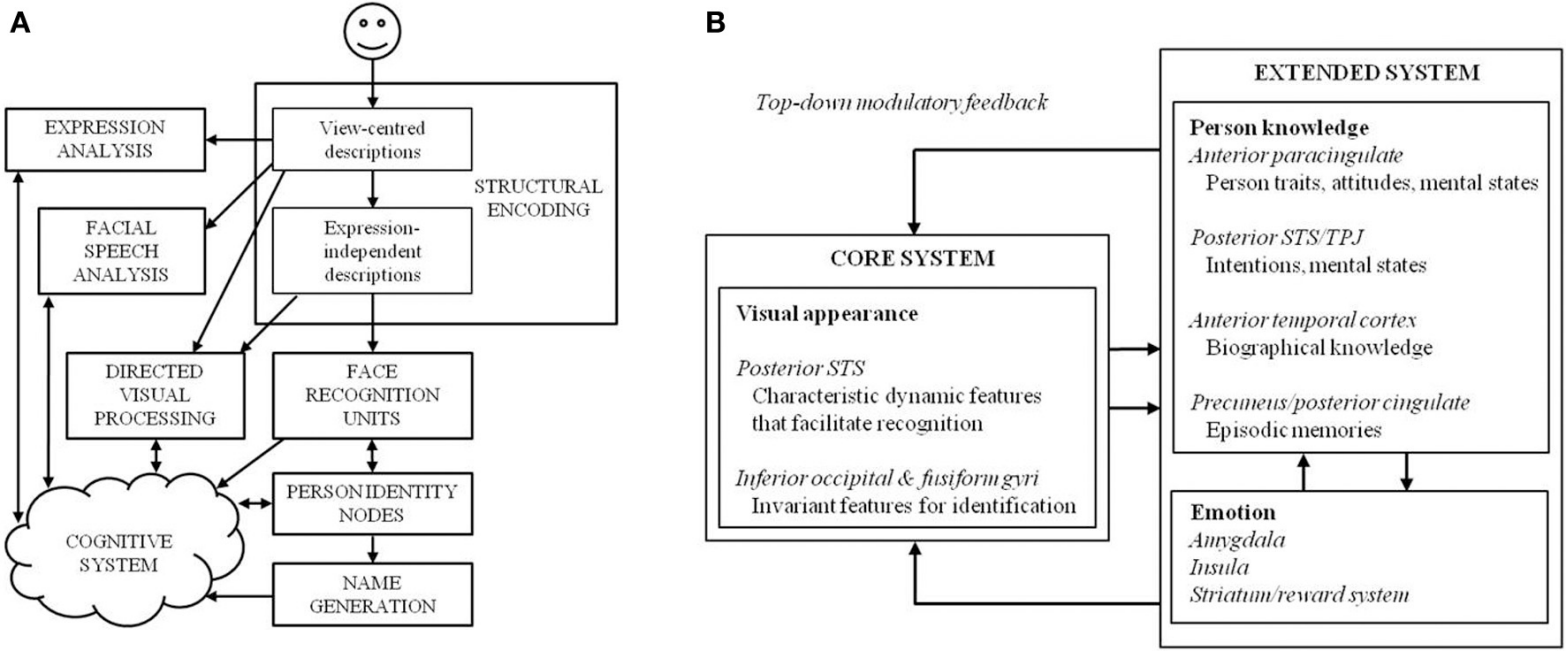
**(A)** The cognitive model of face-processing proposed by Bruce and Young (1986), and **(B)** an adaptation of the distributed model of face-processing proposed by Gobbini and Haxby (2007).

Initial cognitive assessments are required to inform the design of an intervention programme, although previous attempts at cognitive neuropsychological rehabilitation have often failed to follow this principle (Wilson and Patterson, 1990; Hillis, 1993). Fortunately, we have a relatively sophisticated understanding of the cognitive and neural underpinnings of the face-processing system, and dominant models of face recognition have traditionally been used to interpret cases of prosopagnosia and to guide intervention strategy. Traditionally, the face-processing system has been viewed as a sequential and hierarchical multi-process system, where impairment can occur at a variety of stages (Bruce and Young, 1986; see Figure 1). Specifically, an initial stage of early visual analysis is followed by “structural encoding,” where view-centered representations (used to perceive changeable aspects of the face, such as emotional expression) are transformed into viewpoint-independent representations (used to perceive unchangeable aspects of the face—most notably identity). The face recognition units (FRUs) compare all stored representations of familiar faces to an incoming percept. If a match is achieved, access to semantic information is provided by the relevant person identity node (PIN), culminating in retrieval of the person's name. Although these processes are widely distributed across many neural systems that work in concert to process faces, specialized anatomical structures have been identified that largely map onto the functional stages proposed in the cognitive model (Haxby et al., 2000; see Figure 1).

The modular model permits disruption either to specific sub-processes, or to the connections between different units. The sequential nature of the model assumes that processing cannot be continued (at least at an overt level) past a damaged stage. Thus, prosopagnosia may result from three loci of damage within the framework: first, an AP may be unable to construct an adequate percept of a face, which would affect all later stages of processing (i.e., they would be unable to recognize a face as familiar or identify it; e.g., patient HJA: Humphreys and Riddoch, 1987; patient BM: Sergent and Villemure, 1989); second, an AP may be able to achieve a normal face percept but cannot access stored face memories (the FRUs)—in this case, they would be unable to ascertain familiarity or identity (e.g., patient LH: Etcoff et al., 1991; patient NR: De Haan et al., 1992); or third, an AP may be able to perceive faces and make familiarity judgments, but fail to access person-specific information or PINs—in this case, they would achieve a normal face percept and a sense of familiarity with a face, but identification (i.e., access to any semantic information about the person) would remain poor (e.g., patient ME: De Haan et al., 1991a).

In the majority of cases reported in the literature, patients with AP retain the ability to recognize people on the basis of other, non-face cues (e.g., body, voice). In some cases, however, impairments in face recognition are a subset of a more general person recognition problem—this is often associated with damage to the right anterior temporal lobe (Gainotti, 2013). In other words, these cases represent a subtly different type of disorder—one of semantic memory. Various interpretations of the exact nature of semantic disorders of this type exist, including impaired overt access to an output from semantics (Hanley et al., 1989), inability to use a “common access point” to gain semantic information (De Haan et al., 1991a), actual loss of person-based semantic knowledge (Evans et al., 1995; Laws et al., 1995), and damage to a specialized semantic store that contains information about singular objects (Ellis et al., 1989).

It therefore follows that an initial assessment should identify the functional locus of the impairment—be it perceptual, mnemonic, or a more general semantic memory problem—and training should be tailored to that weakness. Several cases in the AP rehabilitation literature demonstrate the importance of tailoring training programmes to the locus of the deficit. Most strikingly, Francis et al. (2002) created a number of therapy tasks tailored to patient NE, who had deficits at both structural and semantic levels, and/or deficits in the access links between structural and semantic knowledge. In three studies, the authors demonstrated that therapy was effective when it emphasized semantic information about people, and linked this knowledge to visual representations (imagery or photographs of faces); whereas therapy directed at processes that were not underpinning the impairment (i.e., name retrieval) was unsuccessful. In another case, Powell et al. (2008) investigated the rehabilitation of face recognition deficits in 20 adults who presented with a broad range of cognitive impairments following brain injury. The participants completed three training programmes targeted at the recognition of unfamiliar faces, comprised of (1) a semantic association technique that provided additional verbal information about faces, (2) caricatured versions of target faces for recognition, and (3) a part-recognition technique that drew participants' attention toward distinctive facial features. The patient group as a whole showed small improvements in each of the three training conditions compared to a control condition where participants were simply exposed to faces. However, when the techniques were applied to a single case of profound acquired prosopagnosia (patient WJ, described in McNeil and Warrington, 1993; see Table 1), little or no improvement was observed following the semantic association and caricaturing programmes, whereas the part-recognition technique yielded 25% greater accuracy than the control condition. This result may be explained by focussing on the functional locus of impairment: WJ was impaired at the level of structural encoding, and relied on a feature-by-feature processing strategy that could be boosted by compensatory training. In some ways this is a surprising finding given that many prosopagnosics adopt this strategy in everyday life, and one might expect that WJ would naturally be using the technique even in the “simple exposure” condition. Nevertheless, this finding suggests not only that part recognition may be an effective method of circumventing damage to the typical face recognition system, but also that training in use of the technique may further boost a compensatory strategy that many individuals with prosopagnosia naturally adopt.

Clearly though, regardless of whether training is targeted at the impairment itself, other influences may prevent training success (e.g., KD, Ellis and Young, 1988). For instance, different levels of impairment may be more or less amenable to treatment: a number of authors have argued that prosopagnosia arising from perceptual deficits is most resistant to treatment and also least likely to show treatment generalization effects (Wilson, 1987; Ellis and Young, 1988; Francis et al., 2002). Polster and Rapcsak (1996) examined the effects of “deep encoding”—that is, incorporating personality judgments or providing names and other semantic information at the point of encoding—in patient RJ. They found that RJ, who showed face perception impairments, did not benefit from “shallow” encoding instructions to focus on facial features, yet performed relatively well with “deep” encoding instructions where he was required to rate faces in terms of their personality traits or was provided with semantic or name information during the study phase. The authors suggest that semantic information may aid recognition memory by establishing additional visually derived and identity-specific semantic codes. However, the gains did not generalize to novel viewpoints of the learned faces, and the authors conclude that the patient simply could not compensate for his inability to construct abstract structural codes that normally allow faces to be recognized from different orientations. Hence, even training in compensatory behavioral mechanisms could not circumvent the severity of the patient's face perception impairment.

While perceptual difficulties may well contribute to intervention success, it is of note that another study failed to rehabilitate an AP adult with higher-order impairments, patient PH. PH had profound face recognition impairments, but was found to display some covert recognition on several behavioral tasks, indicating he had a higher-level impairment affecting the FRUs or PINs, or the connection between them. Based on the knowledge that PH was capable of face recognition on an unconscious level, De Haan et al. (1991b) used a category-presentation method to try to improve the patient's face-processing skills. Specifically, PH was presented with the occupation performed by a set of famous people, and was asked to subsequently recognize their faces. Unfortunately, PH was only successful in recognizing faces from one of the six occupational categories that was used in the study, and the improvement was not maintained in a follow-up test 2 months later. This does not suggest that higher-order impairments cannot be remedied, but it does emphasize that, as discussed above, other factors such as age and lesion severity may contribute to the success of rehabilitation—it is pertinent to note that PH was an adult who had experienced bilateral damage to the temporo-occipital junction, and he did present with some perceptual impairments (see Table 1).

Finally, some cases of AP present with damage to more than one sub-process of the theoretical model. Francis et al. (2002) suggest that, when a patient's deficit is due to multiple impairments, intervention must target each of these in order for improvement to occur. For example, in their investigation described above, the authors found that therapy targeted at only one of NE's deficits (the semantic problem) without considering the other (the prosopagnosia) was ineffective.

### Implications for intervention: compensatory or remedial training?

One of the critical debates in neurorehabilitation is concerned with whether training should encourage the formation of behavioral compensatory mechanisms, or attempt to strengthen normal behavioral mechanisms (remedial training). There has been only one attempt to restore normal processing in a case of AP to date, which unfortunately was not successful (KD, Ellis and Young, 1988). Clearly, no conclusions can be drawn on the utility of remedial methods for acquired cases on a single case alone, particularly given the unusual characteristics of the case (i.e., the age of acquisition, treatment option, and lesion size and location: see section Lesion Size and Location).

While attempts at remedial training are currently very limited, three of the four published studies examining the use of compensatory strategies in AP report some success (see Table 1). It is of note that two of these studies describe individuals with similar perceptual deficits in face-processing, yet found success using different techniques. While Powell et al. (2008) found a benefit of part-based but not semantic encoding for WJ, Polster and Rapcsak (1996) found a greater benefit for semantic or “deep” encoding than part-based encoding for patient RJ. It is unclear why featural and not semantic training helped WJ whereas the reverse pattern was observed in RJ, but these reports suggest both techniques may be beneficial, albeit for different individuals.

Of the studies presented in Table 1, only one of the four compensatory training studies had no effect—the study presented by De Haan et al. (1991b). Pertinently, the patient described in this study differs from those in the other studies, as they had a severe mnemonic rather than perceptual difficulty, and had also suffered bilateral damage. Based on the limited available evidence, compensatory training therefore appears to be more successful in AP than remedial techniques. Yet, further research is clearly required to examine the utility of remedial training in this form of the condition, and to assess which factors may influence the success of various training methods—for example, perhaps remedial training is more effective for patients with unilateral lesions, or for those with mnemonic deficits. Indeed, research into face-name encoding in Alzheimer's disease has had some success with remedial mnemonic techniques such as errorless learning and spaced retrieval (e.g., Haslam et al., 2011), but these techniques have not yet been applied in mnemonic cases of AP.

Understanding the conditions in which remedial techniques are effective is particularly important given that the wider neurorehabilitation literature suggests their benefits are larger than those of behavioral compensation (e.g., Sitzer et al., 2006). Within the AP literature, compensatory techniques show some limitations: NE (Francis et al., 2002) showed significant gains following training, but despite her success in the laboratory, she continued to encounter substantial problems in everyday life. She interpreted this as a case of competing demands—she was using a highly contrived method for remembering and recognizing new people, as well as coping with more general memory deficits. Such instances highlight the limitations of compensatory training, and should remedial training prove effective for at least some cases of AP, this may be a preferable option in terms of outcome.

## Developmental disorders

### DP and neuroplasticity

While we do not yet have a complete understanding of the genetic, neurological, and cognitive underpinnings of DP, it is viewed by most as a parallel disorder to AP. Yet, some caution should be exercised in application of the principles of neurorehabilitation discussed above to the developmental form of the condition. Thomas (2003) notes that developmental disorders represent the limits of plasticity, given that spontaneous reorganization and compensation during the natural developmental process do not overcome whatever abnormalities are underpinning the condition, as they may do following focal damage in the peri- or postnatal period (e.g., Mancini et al., 1994). Granted, it would be very difficult to actually find any cases of spontaneous recovery in DP, and this is further complicated by our limited understanding of the developmental trajectory of the condition and the existence of any early biobehavioral indicators. Nevertheless, the persistence of deficits in developmental disorders suggest atypical limitations on plasticity rather than focal damage, perhaps because disruption to early brain development alters low-level neurocomputational constraints, which prevent certain neural regions from acquiring normal specialized functions (Thomas and Karmiloff-Smith, 2003). It has been suggested that DP can be attributed to a failure to develop the visuo-cognitive mechanisms required for successful face recognition (Susilo and Duchaine, 2013), although it is unclear whether this comes about via genetic influences (Kennerknecht et al., 2006) or unrelated neurological abnormalities (e.g., Behrmann et al., 2007; Garrido et al., 2009). Importantly, while there is some evidence for a genetic factor in DP, Pennington (2001) argues that the correspondence between genes and the complex behavioral phenotypes observed in heterogeneous disorders such as DP is many-to-many rather than one-to-one. Hence, it is unlikely that a specific gene or set of genes exists for certain cognitive functions, including face-processing.

Understanding the underpinnings of DP is an important issue when it comes to the design of intervention programmes: Karmiloff-Smith and colleagues warn that apparently normal behavior in developmental disorders may be achieved by compensatory strategies that obscure underlying atypical processes (Karmiloff-Smith et al., 2002). In the context of face-processing this is evident in Williams Syndrome, a chromosomal disorder where face recognition skills are apparently normal (e.g., Wang et al., 1995), yet are underpinned by poor configural processing mechanisms (Karmiloff-Smith et al., 2004). It is also clear that individuals with DP develop complex and intriguing compensatory strategies that permit them to disguise their face recognition impairment in many real life scenarios (e.g., Yardley et al., 2008), and it remains unclear whether these techniques can sometimes obscure impaired processing strategies on behavioral tests of face and object processing. Thus, an important implication for the design of intervention programmes is that apparently specific cognitive deficits in developmental disorders do not necessarily imply a specific and localized site of neural impairment as has traditionally been observed in cases of adult brain damage.

This latter point has important implications for the notion that training should target the locus of functional impairment (see section Identifying the Functional Impairment). Several authors have attempted to interpret DP within the same theoretical framework that has traditionally been used for AP (e.g., Bruce and Young, 1986), and have used these findings to subsequently inform their rehabilitation programmes (e.g., Brunsdon et al., 2006; Schmalzl et al., 2008). However, some caution should be exercised when applying developmental deficits to adult frameworks of normal functioning. The traditional cognitive neuropsychological approach adopts the logic that implications about cognitive structure can be derived from the patterns of behavioral impairment that are observed in adults with acquired brain damage—for instance, the assumption that particular cognitive systems have modular structures allows for the possibility that highly selective patterns of impairment implicate relative independence of different sub-processes. Interpretation of apparently similar patterns of deficits in developmental disorders is tempting, particularly as one might infer that specific impairments in acquired and developmental cases correspond to acquired damage to a particular module in the former, and failure to develop that module in the latter (notably, Temple, 1997; Temple, offers just such a characterization for cases of DP). Yet, this inference is controversial, and some researchers have argued that development itself violates the basic assumptions of classic cognitive neuropsychological models, and there is no reason to suppose that abnormalities in development lead to the production of a cognitive system that simply maps onto the fully developed system (Bishop, 1997; Karmiloff-Smith, 1997).

**Figure 2:**
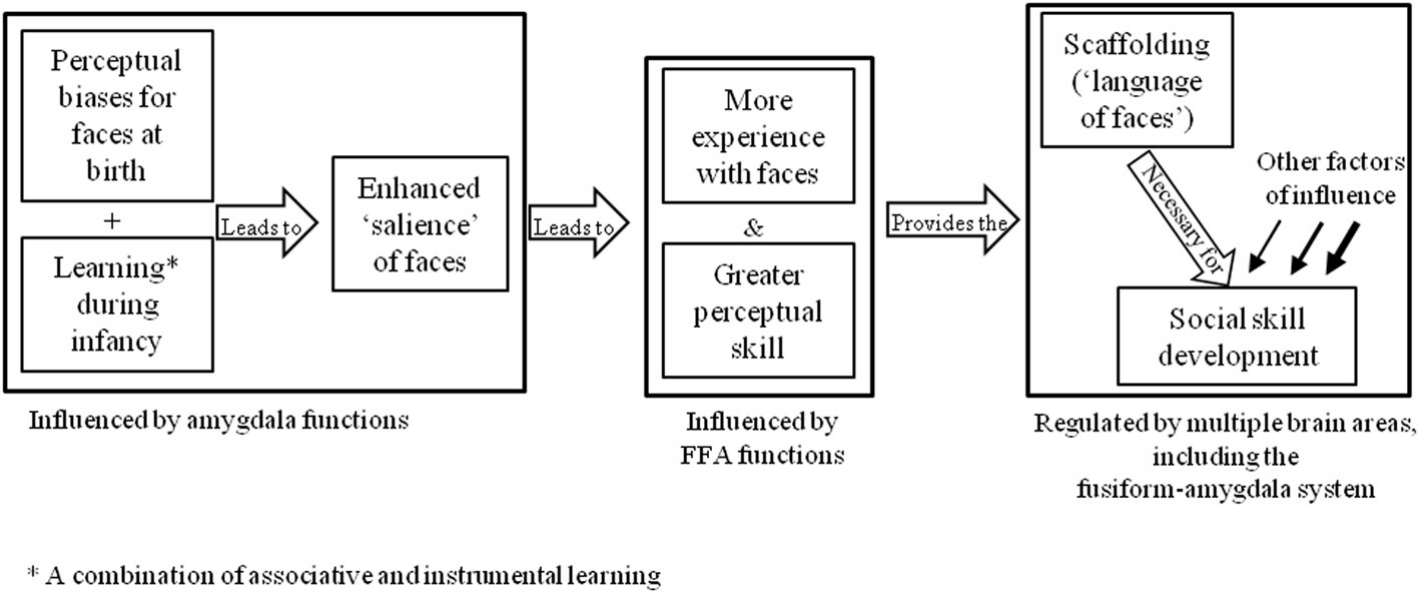
**Schultz's (2005) amygdala/fusiform modulation model**.

Alternative explanations for DP may be found in the neurodevelopmental theories described in section Introduction. For example, one might assume that the basic apparatus for the face-processing system are present, but an abnormality in development has prevented these brain areas from becoming specialized for faces. One theory that adopts this notion is the amygdala/fusiform modulation model (Schultz, 2005), which proposes that the preference for face-like stimuli seen in newborn infants is underpinned by functions in the amygdala that draw attention to social stimuli. This increased social attention is thought to consequently provide the scaffolding that supports social learning and modulates activity in the critical face-processing area of the brain, the fusiform gyrus (see Figure 2). This model has been used to explain the underpinnings of face-processing and socio-emotional deficits in autism spectrum disorder (ASD), based on the premise that faces have less emotional salience for these individuals.

The theory that face-processing deficits in ASD stem from a lack of social interest in faces has informed the development of face training programmes, such as the *Let's Face It* package (Tanaka et al., 2003). *Let's Face It* is a series of computerized games that target the child's ability to attend to faces, in addition to identity and expression recognition skills. Some gains have been noted in ASD participants following participation in the programme (Tanaka et al., 2010), although it is unlikely that similar gains would result in DP given the proposed visuo-cognitive rather than socio-attentional underpinnings of the condition (e.g., Duchaine et al., 2010). Although we do not have a clear understanding of the actual underpinnings and developmental trajectory of DP, the evidence from the ASD literature suggests that intervention can initiate specialization within a crude face-processing system, and that there may be potential for remedial training techniques in developmental conditions.

### Compensatory or remedial training?

The more general neurodevelopmental literature casts doubt on the potential for remedial training in developmental disorders. For instance, Thomas (2003) concludes that only compensatory changes can take place in developmental disorders, as underlying abnormalities are built into the relevant neural structures preventing experience-dependent plasticity. De Haan (2001) presents an example of this argument using a group of individuals with ASD, none of whom could categorically perceive facial expressions. Yet, only those participants with lower IQs appeared to be impaired on an expression-recognition task, indicating that the individuals with higher IQs were using compensatory strategies to achieve good recognition by other means. She therefore allows that there is “a degree of plasticity in the developing system that allows for development of alternative strategies/mechanisms in face-processing” (p. 393), but little to no opportunity for remediation.

In the DP literature, there have been two attempts to improve face recognition via compensatory strategies, and two to remedy normal face-processing strategies (see Table 1). First, Brunsdon et al. (2006) attempted to improve face recognition skills in an eight year-old child (AL), who had problems perceiving and recognizing faces. The researchers gave AL a set of 17 personally known faces (i.e., those of friends and family) to learn on stimuli cards, while his attention was drawn to distinguishing features of the faces. AL continued training until he recognized all the faces in four consecutive sessions, which occurred after 14 sessions within a 1-month period. A similar technique was adopted by Schmalzl et al. (2008), in their work with K, a four-year-old girl with DP. K achieved 100% accuracy in four consecutive sessions after nine attempts at training, and eye movement recordings indicated that she spent a longer time viewing the inner facial features after training. Both children reported benefits to their everyday recognition of the trained faces, although the benefits of training did not generalize to untrained faces in AL (generalization was not tested in K).

On the other hand, DeGutis et al. (2007) described a remedial training programme that suggests normal networks can be strengthened in DP. They report the case of an adult with DP, MZ, who had severe impairments in face perception. The training task was administered over 14 months in two separate intervals. Training required MZ to perform a perceptual classification task repeatedly over large numbers of trials. Specifically, facial stimuli were adjusted to vary in 2 mm increments according to eyebrow height and mouth height. MZ was required to classify each face into one of two categories: those faces with higher eyebrows and lower mouths, and those faces with lower eyebrows and higher mouths. After training, behavioral evidence indicated that MZ's face-processing ability improved on a range of behavioral tasks. However, the most pertinent findings of the study came from changes in neurophysiological measures that were taken before and after training. Specifically, the authors used electroencephalography to investigate whether MZ displayed a selective N170 response for faces compared with watches. Although this face-selective component was not evident before training, its selectivity after training was normal. Further, levels of functional connectivity between key areas of the neurological face-processing system (see Figure 1) were increased after training. The authors suggested the training task was likely successful because it allowed MZ to become sensitive to spacing differences around the eye region and nose/mouth region and encourage her to integrate the spacing of these features into a coherent representation of the face. This gain was specific to training with upright faces: 8000 training trials with inverted faces improved MZ's ability to classify inverted faces but did not improve her performance with upright faces. However, there are some important caveats to these findings. MZ showed limited maintenance of training gains: she reported that the behavioral benefits faded after a few weeks without training, and post-training measures showed that her face-specific N170 had reverted back to its pre-training lack of face sensitivity after 15 weeks without training. Notably though, when the authors attempted to retrain MZ 15 weeks after training stopped, fewer trials were required than in the initial training to restore her improved performance on the assessment tests.

These findings were given weight by DeGutis et al. (2014) who showed that holistic processing improved in 13 out of 24 DPs who completed the same training programme over a 3 week period. Interestingly, the DPs who responded better to training only differed from those who achieved little gains according to the CFMT (a test of face memory: Duchaine and Nakayama, 2006) and not tests of face perception. In fact, the DPs who responded most to training were initially poorer at the CFMT (i.e., their prosopagnosia was more severe), although this comparison was not significant when a *post-hoc* correction was applied.

In sum, while at least some success was achieved in all four DP studies reported to date, it is difficult to draw general conclusions on the utility of each technique, particularly given the differences in age between the participants. The next section evaluates the factors that may have influenced treatment outcome in the studies described above.

### Other influences on treatment outcome in DP

In the AP literature, a number of authors have argued that level of impairment in prosopagnosia is an important factor in treatment outcome, and particularly that prosopagnosia arising from perceptual deficits is more resilient to intervention and generalization (Wilson, 1987; Ellis and Young, 1988; Francis et al., 2002). Although it is currently unclear whether DP can also be partitioned into different functional subtypes, some individuals with DP do appear to present with deficits in face perception, whereas others do not (e.g., Bate et al., 2009). Interestingly, the two compensatory training studies used children who did have impairments in face perception, and while there was little evidence of generalization to other faces (analogous to the findings in the AP literature), the gains did translate to everyday life. These studies demonstrate that, in DP, the recognition of a set of familiar face photographs can be improved with relatively little but precisely targeted training, even in the context of severe face perception impairments. Perhaps more strikingly, everyday gains were also noted in the individual reported by DeGutis et al. (2007), who also had a severe face perception impairment. This finding indicates that it is possible to apply remedial programmes to individuals with perceptual impairments, at least in adults with DP. Critically, DeGutis et al. (2014) found that larger training gains appear to be associated with poorer face recognition performance, and were not related to perceptual abilities.

Given that DeGutis et al.'s (2014) remedial training programme was not successful in all DPs, it is likely that different subtypes of the condition are better suited to particular training methods. As only one (unsuccessful) remedial programme has been trialed with an AP participant, it remains unclear whether (a) DP is simply easier to treat than AP using remedial training, (b) perceptual deficits are not as severe in DP as in AP, (c) the methods used in the DP studies are simply more effective than those employed in the AP studies, or (d) the nature of the lesion in the AP participant precluded any improvement regardless of intervention strategy.

One might also question the influence of age in the DP studies (see section Timing of Injury). From the available evidence it is very difficult to draw any conclusions on the suitability of remedial or compensatory training for different age groups, given the former were only carried in adults, and the latter in children. However, the studies reported by DeGutis and colleagues indicate that plasticity is retained in adult DPs, and provides encouraging evidence for the use of remedial programmes even in adulthood. Whether the same benefits will be exacerbated in children is unknown, but Dalrymple et al. (2012) briefly describe a DP child, TM, for whom remedial training was not successful. She notes several explanations for this, including the severity of his prosopagnosia, the intensity of training, and motivational factors (the training was quite tedious). It is clear that, although successful training strategies are beginning to emerge in adult studies, these strategies will need to be adapted and made age-appropriate for children, even if they target similar mechanisms.

If early intervention is critical in DP (before the development of unhelpful compensatory strategies and the passing of any critical periods), research needs to focus on early detection of the condition. Bradshaw (2001) argues that the consequences of atypical development may not be observable on a behavioral level for some time after they have occurred, indicating that urgent work is required to establish the developmental trajectory of DP, and its biobehavioral markers and risk factors.

## Further considerations of intervention programmes

### Specificity of training

It is clear from the above discussion that the most successful training programmes (whether compensatory or remedial) are those that target the impairment itself. In particular, the studies reported by DeGutis et al. (2007, 2014) indicate that training in holistic processing—a mechanism that is believed to be disrupted in both AP and DP—may be particularly fruitful. Pertinently though, it is possible to target such mechanisms using both facial (e.g., Maurer et al., 2002) and non-facial (e.g., Navon, 1977) stimuli. Such findings have important implications for training, given evidence that intervention using non-facial holistic processing techniques may not be beneficial for individuals with prosopagnosia. For instance, as mentioned in section Compensatory or Remedial Training? training with inverted face stimuli did not improve performance with upright faces in a participant with DP (DeGutis et al., 2007). A similar finding was reported in a study that attempted to train neurotypical participants in holistic processing using inverted faces (Robbins and McKone, 2003). While it is unclear exactly why this effect occurs, it is possible that training with inverted faces simply does not improve holistic processing strategies, and instead encourages processing strategies that are optimal for the recognition of inverted but not upright faces (Farah, 1996; Kanwisher, 2000). Alternatively it may simply be that there is a limit to the amount of transfer that is possible in perceptual learning, and upright faces are just too different from inverted faces for any gains to generalize (Fahle, 2005).

Perhaps the most striking demonstration of the need for face-specific training comes from a study reported by Behrmann et al. (2005). These authors describe the case of SM, a 24 year-old man with visual agnosia and concomitant prosopagnosia following damage to the right anterior and posterior temporal lesions, corpus callosum, and left basal ganglia. The authors trained SM to recognize Greebles (novel objects that require the integration of different “features” composed of complex shapes; Gauthier and Tarr, 1997) over a 31 week period. As has been observed in previous studies (e.g., Gauthier and Tarr, 1997; Duchaine et al., 2004) SM showed a significant improvement in recognizing Greebles that also extended to untrained stimuli and common objects. However, his face recognition skills became even more impaired following training. When this became evident, the authors stopped the training programme and concluded that residual neural tissue with limited capacity may compete for representations. These findings indicate that, at least in the case of holistic processing, any attempts to remediate prosopagnosia must utilize faces in order to be effective.

### Generalization, maintenance and transfer

Failure to elicit treatment generalization both to untreated items and also to alternative versions of the treated items has been common in the treatment of visual recognition difficulties, for both objects and faces (see Riddoch and Humphreys, 1994). In the AP studies that showed some success, there was only evidence of generalization in the study reported by Francis et al. (2002). In fact, these authors concur with Ellis and Young (1988) that level of impairment is an important factor in remediation outcome and particularly findings of generalization. Francis et al. (2002) propose that person-specific generalization in their study within the treated group of photos (i.e., generalization of trained images to other images of the same person) may have been related to the fact that NE did not exhibit perceptual deficits. They propose that failures to achieve this type of generalization in other cases may relate to difficulties earlier in face-processing and particularly at a perceptual level (Ellis and Young, 1988).

However, a different pattern emerges in the DP literature. The one study that assessed generalization of the compensatory training programme within laboratory-based assessments found no evidence of generalization to untrained faces, although AL did show the benefits for different images of the trained faces (Brunsdon et al., 2006). However, response latencies were unusually long in AL, suggesting implementation of the strategy was labored. This observation is akin to the report of NE (Francis et al., 2002), who also received benefits from compensatory training, but found the strategies were often inefficient to implement in everyday life. Nevertheless both AL and K (Schmalzl et al., 2008) reported improved recognition of the trained individuals in everyday life, and the gains were maintained at 3-month and 4-week follow-ups, respectively. K was also described in Wilson et al. (2010) when she was 7.5 years old, and continued maintenance of the gains was reported (but note that the authors suggest K may be on the autism spectrum). These observations suggest that in DP compensatory training may be rapid, suitable for adults and young children, suitable for individuals with perceptual impairments, and the gains may translate to everyday life (but only for trained faces) and be maintained.

On the other hand, the remedial holistic training programme reported by DeGutis et al. (2007, 2014) also generalized to improvements in everyday face recognition (i.e., the gains were not restricted to the faces used in training), as evidenced by self-report diaries kept by the participants. However, MZ showed limited maintenance of training gains (DeGutis et al., 2007), which raises the possibility that while remedial training may bring about greater and more generalized gains, these benefits may quickly fade without continued rehearsal. Furthermore, training in the larger group study was only successful in 13 of the 24 participants, and was not linked to pre-training performance on perceptual tests. This indicates that gains from remedial training can vary significantly between individuals, and a more complex set of factors may influence treatment outcome.

### Individual differences

Much evidence indicates that age may be an important variable in predicting success in neurorehabilitation. Although no clear patterns can currently be seen in the prosopagnosia literature, it is likely that participant age may dictate the choice of training technique. For example, although the DP studies indicate that compensatory training can be effective even in children, the case of TM (Dalrymple et al., 2012) raises the possibility that remedial training techniques are simply not age-appropriate. Given that the broader neurorehabilitation literature suggests that remedial training should be more effective in children, future work needs to develop adaptations of remedial programmes for specific age ranges.

The wider neurorehabilitation literature also suggests that other individual differences can influence intervention outcome, although it is too early to comment on whether these hold true for prosopagnosia. For instance, there is controversial evidence that gender predicts recovery from acquired damage in adulthood (Anderson et al., 2001), as hormones may cause the female brain to develop more rapidly and with a more diffuse organization, perhaps permitting greater plasticity and potential for reorganization of function (Strauss et al., 1992; Kolb, 1995).

In addition, individuals with higher intelligence and superior education are less affected by brain damage (Wilson, 2003), and Anderson et al. (2001) conclude that family function, socioeconomic status, access to rehabilitation, and response to disability all make a powerful contribution to recovery. In the longer-term, it is environmental rather than organic factors that tend to predict recovery from acquired brain damage (e.g., Kolb, 1995). Hence, these factors may influence the outcome of rehabilitation studies, and should be taken into account when evaluating intervention success.

## Future directions

Clearly future work needs to explore both compensatory and remedial training strategies in more depth, and match their suitability to both AP and DP, their potential subtypes, and properties of the individual participant. Future work should also investigate participants' emotional response to interventions—for example, whether training programmes can lead to negative outcomes (e.g., frustration or feelings of low self-worth if they are ineffective), and how these compare to the relatively modest behavioral gains reported to date. Future studies may also move beyond purely behavioral interventions: given huge gains in everyday face recognition have not been reported following any type of training, alternative methodologies may present with more fruitful means of boosting face recognition skills in prosopagnosia. Two methodologies in particular have the potential to supplement face training programmes: intranasal inhalation of oxytocin and non-invasive brain stimulation.

Recent evidence suggests that intranasal inhalation of oxytocin can temporarily improve face recognition skills in both typical participants and those with DP. Oxytocin is a neuropeptide that affects social cognition, potentially by increasing the perceptual salience of social cues (Bartz et al., 2011). Several studies of neurotypical populations have found better memory for faces (but not other, non-social stimuli) following inhalation of oxytocin (Guastella et al., 2008; Savaskan et al., 2008; Rimmele et al., 2009). More notably, a recent study found that participants with DP showed better performance on both a face matching and a face memory task following inhalation of oxytocin, compared with a placebo condition (Bate et al., 2014). Currently it is unclear why people with DP benefit from inhalation of oxytocin. On a neural level, findings from participants with typical face recognition suggest that oxytocin modulates activity in several regions implicated in face processing—namely, the FFA and the amygdala (Domes et al., 2010; Gamer et al., 2010). DPs show structural and connectivity abnormalities in the core face-processing system, around the fusiform and temporal gyri (Garrido et al., 2009) and within the ventro-occipital cortex (Thomas et al., 2009). Therefore, it is possible that oxytocin-related modulation of activity in these areas could underpin increased face recognition performance for the DPs in Bate et al.'s (2014) study. However, further work incorporating neuroimaging of DPs under oxytocin conditions is necessary to explore this possibility.

Inhalation of oxytocin has been found to increase fixations to the eye region of the face in typical participants (Guastella et al., 2008; Gamer et al., 2010). The eye region is considered optimal for face recognition (Peterson and Eckstein, 2012), and several studies have found that DPs spend less time looking at the eye region than typical controls (e.g., Schwarzer et al., 2007). It is possible that oxytocin encouraged DP participants to attend to the eye region more than usual, which may have increased their performance in face-processing tasks. Once again, further work using eye-tracking technology is necessary to explore this possibility. Future work may consider combining inhalation of oxytocin with behavioral training in an attempt to increase or speed up training gains, and/or to extend the benefits of oxytocin inhalation beyond a single session.

Another class of techniques that has been shown to improve face recognition performance, at least temporarily, is non-invasive brain stimulation. There are many types of non-invasive brain stimulation, but three in particular show promise for interventions in prosopagnosia: transcranial electric stimulation (incorporating transcranial direct current stimulation, or tDCS; and transcranial random noise stimulation, or tRNS) and galvanic vestibular stimulation (GVS). In transcranial electric stimulation, a weak current (usually 1–3 mA) is applied to the scalp via electrodes. tDCS involves the use of a constant current. Areas under the anode exhibit cortical excitability, whereas areas under the cathode show the opposite effect (Paulus, 2011). tDCS has been shown to improve performance in typical participants in a range of cognitive tasks, from low-level vision, executive functioning, memory, and language (Kuo and Nitsche, 2012). Notably, tDCS has also been used in stroke patients (generally those with aphasia), and, in concert with cognitive training, has been shown to improve speech and naming abilities (see Krause and Cohen Kadosh, 2013, for a review). This may occur because tDCS facilitates compensation in non-damaged regions, reduces activation in non-damaged regions that may inhibit activation in or around lesioned areas, or increases residual output of partially damaged areas (Cohen Kadosh, 2013). In other words, tDCS may be useful in conjunction with both remedial and compensatory training strategies, but choice of strategy and stimulation site (lesion area/contralateral lesion area) could vary patient-to-patient, depending on the site and extent of damage. To date, tDCS has not been applied to prosopagnosia, or in face perception tasks in typical participants. However, Ross et al. (2010) found that anodal tDCS over the right anterior temporal lobe significantly improved name recall for famous faces in a group of young adults with typical face recognition, indicating that anterior temporal tDCS may be useful in mnemonic cases of AP or DP.

tRNS involves the use of a current that changes several hundred times per second, taking its value from a random noise distribution centered around 0 (Paulus, 2011). Because the current oscillates between the two electrodes, there is no anode or cathode, and the areas under both electrodes show enhanced cortical excitability (Cohen Kadosh, 2013). Like tDCS, tRNS has been shown to improve cognitive abilities in a range of domains, including motor and perceptual learning (Terney et al., 2008; Fertonani et al., 2011). tRNS also shows long-term effects: when combined with 5 days of cognitive training for numerosity or mental calculation, stimulation resulted in increased training gains that remained evident between 16 weeks and 6 months later (Cappelletti et al., 2013; Snowball et al., 2013). Like tDCS, tRNS has not been applied in AP or DP as yet. However, evidence from training studies in other domains suggests that combining cognitive training (such as the techniques used by DeGutis et al., 2014) with tRNS may enhance its effects, although work is needed to clarify which combination of training task and stimulation site is effective in various types of prosopagnosia.

GVS resembles tDCS of the vestibular nerve—electrodes are placed on the mastoid bones, which stimulates the vestibular nerve and, in turn, all vestibular relay stations upstream. fMRI studies have revealed that GVS activates a wide range of cortical areas including several associated with face-processing (e.g., the superior temporal gyrus and temporo-parietal cortex; Bense et al., 2001). Only one study has examined GVS in face recognition: Wilkinson et al. (2005) applied GVS to patient RC, who acquired prosopagnosia following damage to the right temporal lobe (amongst other areas). Short sessions of GVS improved RC's face discrimination performance to above-chance levels. However, the discrimination task was not strictly identity-matching—RC was required to choose a face that did not have its eyes and mouth inverted, rather than to choose between two typical faces. As such, it is difficult to say whether the stimulation simply improved detection of abnormalities in a face, or whether the effects would carry over to other face processing tasks (e.g., face memory). Once again, further work is necessary to confirm whether GVS may also be beneficial for DPs, or in other cases of AP with different lesions or functional profiles.

## Summary

In sum, while there have been few attempts to improve face recognition skills in either AP or DP, some tentative conclusions can be drawn from the available data and the wider neurorehabilitation literature. First, there is evidence to suggest that both forms of the condition respond to compensatory training, and that some adults with DP benefit from remedial training (although currently it is unclear precisely why some participants show benefits, whereas others do not). It is also unclear whether remedial programmes may be useful in AP, and in children with DP. While the benefits of compensatory training programmes appear to be that they are suitable for both adults and children and their gains are more long-lasting, they also promote more labored processing strategies that are less likely to generalize to the recognition of untrained faces. On the other hand, remedial training techniques may promote more efficient “normal” processing strategies that are more likely to generalize to untrained faces, yet it takes more training to achieve these gains and they require continued rehearsal.

Given there have been very few studies in this area, further research into the duration, maintenance, and long-term benefits of remedial and compensatory training are necessary. It is likely that the suitability of these programmes for different individuals will have a complex interaction with age, the type of injury in acquired cases, the severity and nature of the prosopagnosia, and other environmental influences. In any case, gains are likely to be mild-to-moderate, and the utility of alternative methodologies (i.e., oxytocin inhalation or brain stimulation) should be considered. It is important to note that use of these techniques is in its infancy, and while single applications may bring about short-term gains in face recognition skills, there are likely to be significant safety considerations associated with everyday application of the techniques. Alternatively, performance of remedial training under oxytocin or stimulation conditions may bring about larger and longer-term benefits than the behavioral programme alone. Future work using more systematic methods and larger participant groups is clearly required, and in the case of DP, there is an urgent need to develop early detection and remediation tools for children in order to optimize intervention outcome.

### Conflict of interest statement

The authors declare that the research was conducted in the absence of any commercial or financial relationships that could be construed as a potential conflict of interest.

## References

[B1] Anderson V. Northam E. Hendy J. Wrennall J. (2001). Developmental Neuropsychology: A Clinical Approach. Hove: Psychology Press

[B2] Arnott S. R. Heywood C. A. Kentridge R. W. Goodale M. A. (2008). Voice recognition and the posterior cingulate: an fMRI study of prosopagnosia. J. Neuropsychol. 2, 269–286 10.1348/174866407X246131 19334314

[B3] Ballantyne A. O. Trauner D. A. (1999). Facial recognition in children after perinatal stroke. Neuropsychiatry Neuropsychol. Behav. Neurol. 12, 82–87 10223254

[B4] Barton J. J. S. (2008). Structure and function in acquired prosopagnosia: lessons from a series of 10 patients with brain damage. J. Neuropsych. 2, 197–225 10.1348/174866407X214172 19334311

[B5] Barton J. J. S. Press D. Z. Keenan J. P. O'Connor M. (2002). Lesions of the fusiform face area impair perception of facial configuration in prosopagnosia. Neurology 58, 71–78 10.1212/WNL.58.1.71 11781408

[B6] Bartz J. A. Zaki J. Bolger N. Ochsner K. N. (2011). Social effects of oxytocin in humans: context and person matter. Trends Cogn. Sci. 15, 301–309 10.1016/j.tics.2011.05.002 21696997

[B7] Bate S. Cook S. (2012). Covert recognition relies on affective valence in developmental prosopagnosia: evidence from the skin conductance response. Neuropsychology 26, 670–674 10.1037/a0029443 22823135

[B8] Bate S. Cook S. J. Duchaine B. Tree J. J. Burns E. J. Hodgson T. L. (2014). Intranasal inhalation of oxytocin improves face processing in developmental prosopagnosia. Cortex 50, 55–63 10.1016/j.cortex.2013.08.006 24074457

[B9] Bate S. Haslam C. Jansari A. Hodgson T. L. (2009). Covert face recognition relies on affective valence in congenital prosopagnosia. Cogn. Neuropsychol. 26, 391–411 10.1080/02643290903175004 19693716

[B10] Behrmann M. Avidan G. Gao F. Black S. (2007). Structural imaging reveals anatomical alterations in inferotemporal cortex in congenital prosopagnosia. Cereb. Cortex 17, 2354–2363 10.1093/cercor/bhl144 17218483

[B11] Behrmann M. Marotta J. Gauthier I. Tarr M. J. McKeeff T. J. (2005). Behavioral change and its neural correlates in prosopagnosia after expertise training. J. Cogn. Neurosci. 17, 554–568 10.1162/0898929053467613 15829077

[B12] Bense S. Stephan T. Yousry T. A. Brandt T. Dieterich M. (2001). Multisensory cortical signal increases and decreases during vestibular galvanic stimulation (fMRI). J. Neurophysiol. 85, 886–899 11160520 10.1152/jn.2001.85.2.886

[B13] Bhatt R. S. Bertin E. Hayden A. Reed A. (2005). Face processing in infancy: developmental changes in the use of different kinds of relational information. Child Dev. 76, 169–181 10.1111/j.1467-8624.2005.00837.x 15693765

[B14] Biernaskie J. Chernenko G. Corbett D. (2004). Efficacy of rehabilitative experience declines with time after focal ischemic brain injury. J. Neurosci. 24, 1245–1254 10.1523/JNEUROSCI.3834-03.2004 14762143 PMC6793570

[B15] Bishop D. V. M. (1997). Cognitive neuropsychology and developmental disorders: uncomfortable bedfellows. Q. J. Exp. Psychol. 50, 899–923 10.1080/713755740 9450382

[B16] Boeri R. Salmaggi A. (1994). Prosopagnosia: commentary. Curr. Opin. Neurol. 7, 61–64 10.1097/00019052-199402000-00011 8173680

[B17] Boutsen L. Humphreys G. W. (2002). Face context interferes with local part processing in a prosopagnosia patient. Neuropsychologia 40, 2305–2313 10.1016/S0028-3932(02)00088-X 12417460

[B18] Bowles D. C. McKone E. Dawel A. Duchaine B. C. Palermo R. Schmalzl L. (2009). Diagnosing prosopagnosia: effects of ageing, sex, and participant-stimulus ethnic match on the Cambridge Face Memory Test and Cambridge Face Perception Test. Cogn. Neuropsychol. 26, 423–455 10.1080/02643290903343149 19921582

[B19] Bradshaw J. L. (2001). Developmental Disorders of the Frontostriatal System: Neuropsychological, Neuropsychiatric, and Evolutionary Perspectives. Hove: Psychology Press

[B20] Brown C. E. Aminoltejari K. Erb H. Winship I. R. Murphy T. H. (2009). *In vivo* voltage-sensitive dye imaging in adult mice reveals that somatosensory maps lost to stroke are replaced over weeks by new structural and functional circuits with prolonged modes of activation within both the peri-infarct zone and distant sites. J. Neurosci. 29, 1719–1734 10.1523/JNEUROSCI.4249-08.2009 19211879 PMC6666293

[B21] Bruce V. Young A. (1986). Understanding face recognition. Br. J. Psychol. 77, 305–327 10.1111/j.2044-8295.1986.tb02199.x 3756376

[B22] Brunsdon R. Coltheart M. Nickels L. Joy P. (2006). Developmental prosopagnosia: a case analysis and treatment study. Cogn. Neuropsychol. 23, 822–840 10.1080/02643290500441841 21049355

[B23] Cappelletti M. Gessaroli E. Hithersay R. Mitolo M. Didino D. Kanai R. (2013). Transfer of cognitive training across magnitude dimensions achieved with concurrent brain stimulation of the parietal lobe. J. Neurosci. 33, 14899–14907 10.1523/JNEUROSCI.1692-13.2013 24027289 PMC3771029

[B24] Carmichael S. T. Archibeque I. Luke L. Nolan T. Momiy J. Li S. (2005). Growth-associated gene expression after stroke: evidence for a growth-promoting region in peri-infarct cortex. Exp. Neurol. 193, 291–311 10.1016/j.expneurol.2005.01.004 15869933

[B25] Chan D. Anderson V. Pijnenburg Y. Whitwell J. Barnes J. Scahill R. (2009). The clinical profile of right temporal lobe atrophy. Brain 132, 1287–1298 10.1093/brain/awp037 19297506

[B26] Cohen L. B. Cashon C. H. (2001). Do 7-month-old infants process independent features of facial configurations? Infant Child Dev. 10, 83–92 10.1002/icd.250.abs

[B27] Cohen Kadosh K. Cohen Kadosh R. Dick F. Johnson M. H. (2011). Developmental changes in effective connectivity in the emerging core face network. Cereb. Cortex 21, 1389–1394 10.1093/cercor/bhq215 21045001 PMC3094719

[B28] Cohen Kadosh K. Johnson M. H. Dick F. Cohen Kadosh R. Blakemore S. (2013). Effects of age, task performance, and structural brain development on face processing. Cereb. Cortex 23, 1630–1642 10.1093/cercor/bhs150 22661406 PMC3446867

[B29] Cohen Kadosh R. (2013). Using transcranial electrical stimulation to enhance cognitive functions in the typical and atypical brain. Trans. Neurosci. 4, 20–33 10.2478/s13380-013-0104-723467363

[B30] Crane J. Milner B. (2002). Do I know you? Face perception and memory in patients with selective amygdalo-hippocampectomy. Neuropsychologia 40, 530–538 10.1016/S0028-3932(01)00131-2 11749983

[B31] Crookes K. McKone E. (2009). Early maturity of face recognition: no childhood development of holistic processing, novel face encoding, or face-space. Cognition 111, 219–247 10.1016/j.cognition.2009.02.004 19296930

[B32] Dalrymple K. A. Corrow S. Yonas A. Duchaine B. (2012). Developmental prosopagnosia in childhood. Cogn. Neuropsychol. 29, 393–418 10.1080/02643294.2012.722547 23140142 PMC4452128

[B33] Damasio A. R. Damasio H. Van Hoesen G. W. (1982). Prosopagnosia: anatomic basis and behavioural mechanisms. Neurology 32, 331–341 10.1212/WNL.32.4.331 7199655

[B34] DeGutis J. Cohan S. Nakayama K. (2014). Holistic face training enhances face processing in developmental prosopagnosia. Brain 137, 1781–1798 10.1093/brain/awu062 24691394 PMC4032098

[B35] DeGutis J. M. Bentin S. Robertson L. C. D'Esposito M. (2007). Functional plasticity in ventral temporal cortex following cognitive rehabilitation of a congenital prosopagnosic. J. Cogn. Neurosci. 19, 1790–1802 10.1162/jocn.2007.19.11.1790 17958482

[B36] De Haan E. H. Bauer R. M. Grève K. W. (1992). Behavioural and physiological evidence for covert face recognition in a prosopagnosic patient. Cortex 28, 77–95 10.1016/S0010-9452(13)80167-0 1572175

[B37] De Haan E. H. Young A. W. Newcombe F. (1991a). A dissociation between the sense of familairity and access to smenatic information concerning people. Eur. J. Cogn. Psychol. 3, 51–67

[B38] De Haan E. H. Young A. W. Newcombe F. (1991b). Covert and overt recognition in prosopagnosia. Brain 114, 2575–2591 1782532 10.1093/brain/114.6.2575

[B39] De Haan M. (2001). The neuropsychology of face processing during infancy and childhood, in Handbook of Developmental Cognitive Neuroscience, eds Nelson C. A. Luciana M. (Cambridge, MA: MIT Press), 381–398

[B40] de Heering A. Houthuys S. Rossion B. (2007). Holistic face processing is mature at 4 years of age: evidence from the composite face effect. J. Exp. Child Psychol. 96, 57–70 10.1016/j.jecp.2006.07.001 17007869

[B41] de Heering A. Rossion B. (2008). Prolonged visual experience in adulthood modulates holistic face perception. PLoS ONE 3:e2317 10.1371/journal.pone.0002317 18509528 PMC2386412

[B42] De Renzi E. Perani D. Carlesimo G. A. Silveri M. C. Fazio F. (1994). Prosopagnosia can be associated with damage confined to the right hemisphere. An MRI and PET study and a review of the literature. Neuropsychologia 32, 893–902 10.1016/0028-3932(94)90041-8 7969865

[B43] De Renzi E. Zambolin A. Crisi G. (1987). The pattern of neuropsychological impairment associated with left posterior cerebral artery infarcts. Brain 110, 1099–1116 10.1093/brain/110.5.1099 3676694

[B44] De Schonen S. Mancini J. Camps R. Maes E. Laurent A. (2005). Early brain lesions and face-processing development. Dev. Psychobiol. 46, 184–208 10.1002/dev.20054 15772971

[B45] Domes G. Lischke A. Berger C. Grossmann A. Hauenstein K. Heinrichs M. (2010). Effects of intranasal oxytocin on emotional face processing in women. Psychoneuroendocrinology 35, 83–93 10.1016/j.psyneuen.2009.06.016 19632787

[B46] Duchaine B. Murray H. Turner M. White S. Garrido L. (2010). Normal social cognition in developmental prosopagnosia. Cogn. Neuropsychol. 25, 1–15 10.1080/02643291003616145 20191404

[B47] Duchaine B. C. Dingle K. Butterworth E. Nakayama K. (2004). Normal Greeble learning in a severe case of developmental prosopagnosia. Neuron 43, 469–473 10.1016/j.neuron.2004.08.006 15312646

[B48] Duchaine B. C. Nakayama K. (2005). Dissociations of face and object recognition in developmental prosopagnosia. J. Cogn. Neurosci. 17, 249–261 10.1162/0898929053124857 15811237

[B49] Duchaine B. C. Nakayama K. (2006). The cambridge face memory test: results for neurologically intact individuals and an investigation of its validity using inverted face stimuli and prosopagnosic subjects. Neuropsychologia 44, 576–585 10.1016/j.neuropsychologia.2005.07.001 16169565

[B50] Elbert T. Heim S. Rockstroh B. (2001). Neural plasticity and development, in Handbook of Developmental Cognitive Neuroscience, eds Nelson C. A. Luciana M. (Cambridge, MA: MIT Press), 191–202

[B51] Elliott E. S. Wills E. J. Goldstein A. G. (1973). The effects of discrimination training on the recognition of White and Oriental faces. Bull. Psychon. Soc. 2, 71–73 10.3758/BF03327717

[B52] Ellis A. W. Young A. W. Critchley E. M. R. (1989). Loss of memory for people following temporal lobe damage. Brain 112, 1469–1483 10.1093/brain/112.6.1469 2597991

[B53] Ellis H. D. Shepherd J. W. Davies G. M. (1979). Identification of familiar and unfamiliar faces from internal and external features: some implications for theories of face recognition. Perception 8, 431–439 10.1068/p080431 503774

[B54] Ellis H. D. Young A. W. (1988). Training in face-processing skills for a child with acquired prosopagnosia. Dev. Neuropsychol. 4, 283–294 10.1080/8756564880954041218720102

[B55] Etcoff N. L. Freeman R. Cave K. R. (1991). Can we lose memories of faces? Content specificity and awareness in a prosopagnosic. J. Cogn. Neurosci. 3, 25–41 10.1162/jocn.1991.3.1.25 23964803

[B56] Evans J. J. Heggs A. J. Antoun N. Hodges J. R. (1995). Progressive prosopagnosia associated with selective right temporal lobe atrophy: a new syndrome? Brain 118, 1–13 7894996 10.1093/brain/118.1.1

[B57] Fahle M. (2005). Perceptual learning: specificity vs. generalization. Curr. Opin. Neurobiol. 15, 154–160 10.1016/j.conb.2005.03.010 15831396

[B58] Farah M. (1996). Is face recognition “special”? Evidence from neuropsychology. Behav. Brain Res. 76, 181–189 10.1016/0166-4328(95)00198-0 8734052

[B59] Fertonani A. Pirulli C. Miniussi C. (2011). Random noise stimulation improves neuroplasticity in perceptual learning. J. Neurosci. 31, 15416–15423 10.1523/JNEUROSCI.2002-11.2011 22031888 PMC6703532

[B60] Fine D. R. (2012). A life with prosopagnosia. Cogn. Neuropsychol. 29, 354–359 10.1080/02643294.2012.736377 23186078

[B61] Francis D. R. Riddoch M. J. Humphreys G. W. (2002). “Who's that girl?” - Prosopagnosia, person-based semantic disorder, and the reacquisition of face identification ability. Neuropsychol. Rehabil. 12, 1–26 10.1080/09602010143000158

[B62] Gainotti G. (2013). Is the right anterior temporal variant of prosopagnosia a form of “associative prosopagnosia” or a form of “multimodal person recognition disorder”? Neuropsychol. Rev. 23, 99–110 10.1007/s11065-013-9232-7 23579426

[B63] Gainotti G. Marra C. (2011). Differential contribution of right and left temporo-occipital and anterior temporal lesions to face recognition disorders. Front. Hum. Neurosci. 5:55 10.3389/fnhum.2011.00055 21687793 PMC3108284

[B64] Gamer M. Zurowski B. Buchel C. (2010). Different amygdala subregions mediate valence-related and attentional effects of oxytocin in humans. Proc. Natl. Acad. Sci. U.S.A. 107, 9400–9405 10.1073/pnas.1000985107 20421469 PMC2889107

[B65] Garrido L. Furl N. Draganski B. Weiskopf N. Stevens J. Tan G. C. (2009). Voxel-based morphometry reveals reduced grey matter volume in the temporal cortex of developmental prosopagnosics. Brain 132, 3443–3455 10.1093/brain/awp271 19887506 PMC2792372

[B66] Gathers A. D. Bhatt R. Corbly C. R. Farley A. B. Joseph J. E. (2004). Developmental shifts in cortical loci for face and object recognition. Neuroreport 15, 1549–1553 10.1097/01.wnr.0000133299.84901.86 15232281 PMC4522001

[B67] Gauthier I. Tarr M. J. (1997). Becoming a “Greeble” expert: exploring the face recognition mechanism. Vis. Res. 37, 1673–1682 10.1016/S0042-6989(96)00286-6 9231232

[B68] Germine L. T. Duchaine B. Nakayama K. (2010). Where cognitive development and aging meet: face learning ability peaks after age 30. Cognition 118, 201–210 10.1016/j.cognition.2010.11.002 21130422

[B69] Gloning I. Gloning K. Hoff H. (1967). On optic hallucinations: a study based on 241 patients with lesions of the occipital lobe and its surrounding regions verified by autopsy or surgery. Wiener Zeitschrift für Nervenheilkunde und deren Grenzgebiete 25, 1–19 4229655

[B70] Glowic C. Violon A. (1981). A case of regressive prosopagnosia (author's translation). Acta Neurol. Belg. 81, 86–97 7234323

[B71] Gobbini M. I. Haxby J. V. (2007). Neural systems for recognition of familiar faces. Neuropsychologia 45, 32–41 10.1016/j.neuropsychologia.2006.04.015 16797608

[B72] Guastella A. J. Mitchell P. B. Dadds M. R. (2008). Oxytocin increases gaze to the eye region of human faces. Biol. Psychiatry 63, 3–5 10.1016/j.biopsych.2007.06.026 17888410

[B74] Hancock K. J. Rhodes G. (2008). Contact, configural coding and the other-race effect in face recognition. Br. J. Psychol. 99, 45–56 10.1348/000712607X199981 17535471

[B75] Hanley J. R. Young A. W. Pearson N. A. (1989). Defective recognition of familiar people. Cogn. Neuropsychol. 6, 179–210 10.1080/02643298908253418

[B76] Haslam C. Hodder K. I. Yates P. J. (2011). Errorless learning and spaced retrieval: how do these methods fare in healthy and clinical populations? J. Clin. Exp. Neuropsychol. 33, 432–447 10.1080/13803395.2010.533155 21229436

[B77] Haxby J. V. Hoffman E. A. Gobbini M. I. (2000). The distributed human neural system for face perception. Trends Cogn. Sci. 4, 223–233 10.1016/S1364-6613(00)01482-0 10827445

[B78] Hayden A. Bhatt R. S. Reed A. Corbly C. R. Joseph J. E. (2007). The development of expert face processing: are infants sensitive to normal differences in second-order relational information? J. Exp. Child Psychol. 97, 85–98 10.1016/j.jecp.2007.01.004 17339043

[B79] Hécaen H. Angelergues R. (1962). Agnosia for faces (prosopagnosia). Arch. Neurol. 7, 92–100 10.1001/archneur.1962.04210020014002 13905818

[B80] Hessen E. Anderson V. Nestvold K. (2007). Neuropsychological function 23 years after mild traumatic brain injury: a comparison of outcome after pediatric and adult head injuries. Brain Injury 21, 963–979 10.1080/02699050701528454 17729049

[B81] Hillis A. E. (1993). The role of models of language processing in rehabilitation of language impairments. Aphasiology 7, 5–26 10.1080/02687039308249497

[B82] Humphreys G. W. Riddoch M. J. (1987). To See But Not To See: A Case Study of Visual Agnosia. London: Lawrence Erlbaum

[B83] Huttenlocher P. R. (2002). Neural Plasticity: The Effects of Environment on the Development of the Cerebral Cortex. Cambridge, MA: Harvard University Press

[B84] Huttenlocher P. R. Dabholkar A. S. (1997). Regional differences in synaptogenesis in human cerebral cortex. J. Comp. Neurol. 387, 167–178 10.1002/(SICI)1096-9861(19971020)387:2%3C167::AID-CNE1%3E3.0.CO;2-Z 9336221

[B85] Josephs K. A. (2007). Frontotemporal lobar degeneration. Neurol. Clin. 25, 683–696 10.1016/j.ncl.2007.03.005 17659185 PMC2702867

[B86] Joubert S. Felician O. Barbeau E. Sontheimer A. Barton J. J. Ceccaldi M. (2003). Impaired configurational processing in a case of progressive prosopagnosia associated with predominant right temporal lobe atrophy. Brain 126, 2537–2550 10.1093/brain/awg259 14506066

[B87] Kanwisher N. (2000). Domain specificity in face perception. Nat. Neurosci. 3, 759–763 10.1038/77664 10903567

[B88] Karmiloff-Smith A. (1997). Crucial differences between developmental cognitive neuroscience and adult neuropsychology. Dev. Neuropsychol. 13, 513–524 10.1080/87565649709540693

[B89] Karmiloff-Smith A. Scerif G. Thomas M. S. C. (2002). Different approaches to relating genotype to phenotype in developmental disorders. Dev. Psychobiol. 40, 311–322 10.1002/dev.10035 11891641

[B90] Karmiloff-Smith A. Thomas M. Annaz D. Humphreys K. Ewing S. Brace N. (2004). Exploring the Williams syndrome face processing debate: the importance of building developmental trajectories. J. Child Psychol. Psych. 45, 1258–1274 10.1111/j.1469-7610.2004.00322.x 15335346

[B91] Katz D. I. Alexander M. P. (1994). Traumatic brain injury: predicting course of recovery and outcome for patients admitted to rehabilitation. Arch. Neurol. 51, 661–670 10.1001/archneur.1994.00540190041013 8018038

[B92] Kelly D. J. Quinn P. C. Slater A. Lee K. Ge L. Pascalis O. (2007). The other-race effect develops during infancy: evidence of perceptual narrowing. Psychol. Sci. 18, 1084–1089 10.1111/j.1467-9280.2007.02029.x 18031416 PMC2566514

[B93] Kelly D. J. Quinn P. C. Slater A. M. Lee K. Gibson A. Smith M. (2005). Three-month-olds, but not newborns, prefer own-race faces. Dev. Sci. 8, F31–F36 10.1111/j.1467-7687.2005.0434a.x 16246233 PMC2566511

[B94] Kennerknecht I. Grueter T. Welling B. Wentzek S. Horst J. Edwards S. (2006). First report of prevalence of non-syndromic hereditary prosopagnosia (HPA). Am. J. Med. Genet. 140, 1617–1622 10.1002/ajmg.a.31343 16817175

[B95] Kolb B. (1995). Brain Plasticity and Behavior. Hillsdale, NJ: Lawrence Erlbaum Associates, Inc

[B96] Krause B. Cohen Kadosh R. (2013). Can transcranial electric stimulation improve learning difficulties in atypical brain development? A future possibility for cognitive training. Dev. Cogn. Neurosci. 6, 176–194 10.1016/j.dcn.2013.04.001 23770059 PMC4064117

[B97] Kuefner D. Macchi Cassia V. Picozzi M. Bricolo E. (2008). Do all kids look alike? Evidence for an other-age effect in adults. J. Exp. Psychol. Hum. Percept. Perform. 34, 811–817 10.1037/0096-1523.34.4.811 18665727

[B98] Kuo M.-F. Nitsche M. A. (2012). Effects of transcranial electrical stimulation on cognition. Clin. EEG Neurosci. 43, 192–199 10.1177/1550059412444975 22956647

[B99] Lang N. Baudewig J. Kallenberg K. Antal A. Happe S. Dechent P. (2006). Transient prosopagnosia after ischemic stroke. Neurology 66, 916 10.1212/01.wnl.0000203113.12324.57 16567712

[B100] Laws K. R. McKenna P. J. McCarthy R. A. (1995). Delusions about people. Neurocase 1, 349–362 10.1080/13554799508402379

[B101] Le Grand R. Mondloch C. J. Maurer D. Brent H. P. (2001). Early visual experience and face processing. Nature 410:890 10.1038/35073749 11309606

[B102] Le Grand R. Mondloch C. J. Maurer D. Brent H. P. (2004). Impairment in holistic face processing following early visual deprivation. Psycholol. Sci. 15, 762–768 10.1111/j.0956-7976.2004.00753.x 15482448

[B103] Lye T. C. Shores E. A. (2000). Traumatic brain injury as a risk factor for Alzheimer's disease: a review. Neuropsychol. Rev. 10, 115–129 10.1023/A:1009068804787 10937919

[B104] Macchi Cassia V. Picozzi M. Kuefner D. Bricolo E. Turati C. (2009a). Holistic processing for faces and cars in preschool-aged children and adults: evidence from the composite effect. Dev. Sci. 12, 236–248 10.1111/j.1467-7687.2008.00765.x 19143797

[B105] Macchi Cassia V. M. Picozzi M. Kuefner D. Casati M. (2009b). Why mix-ups don't happen in the nursery: evidence for an experience-based interpretation of the other-age effect. Q. J. Exp. Psychol. 62, 1099–1107 10.1080/17470210802617654 19142827

[B106] Malone D. R. Morris H. H. Kay M. C. Levin H. S. (1982). Prosopagnosia: a double dissociation between the recognition of familiar and unfamiliar faces. J. Neurol. Neurosurg. Psychiatry 45, 820–822 10.1136/jnnp.45.9.820 7131015 PMC491564

[B107] Malpass R. S. Kravitz J. (1969). Recognition for faces of own and other race. J. Pers. Soc. Psychol. 13, 330–334 10.1037/h0028434 5359231

[B108] Mancini J. de Schonen S. Deruelle C. Massoulier A. (1994). Face recognition in children with early right or left brain damage. Dev. Med. Child Neurol. 36, 156–166 10.1111/j.1469-8749.1994.tb11824.x 8132126

[B109] Mattson A. J. Levin H. S. Grafman J. (2000). A case of prosopagnosia following moderate closed head injury with left hemisphere focal lesion. Cortex 36, 125–137 10.1016/S0010-9452(08)70841-4 10728902

[B110] Maurer D. Le Grand R. Mondloch C. J. (2002). The many faces of configural processing. Trends Cogn. Sci. 6, 255–260 10.1016/S1364-6613(02)01903-4 12039607

[B111] McKone E. Boyer B. L. (2006). Sensitivity of 4-year-olds to featural and second-order relational changes in face distinctiveness. J. Exp. Child Psychol. 94, 134–162 10.1016/j.jecp.2006.01.001 16483596

[B112] McKone E. Brewer J. L. MacPherson S. Rhodes G. Hayward W. G. (2007). Familiar other-race faces show normal holistic processing and are robust to perceptual stress. Perception 36, 224–248 10.1068/p5499 17402665

[B113] McMonangle P. Deering F. Berliner Y. Kertesz A. (2006). The cognitive profile of posterior cortical atrophy. Neurology 66, 331–338 10.1212/01.wnl.0000196477.78548.db 16476930

[B114] McNeil J. E. Warrington E. K. (1993). Prosopagnosia—a face-specific disorder. Q. J. Exp. Psychol. 46A, 1–10 10.1080/14640749308401064 8446761

[B115] Meissner C. A. Brigham J. C. (2001). Thirty years of investigating the own-race bias in memory for faces: a meta-analytic review. Psychol. Public Policy Law 7, 3–35 10.1037/1076-8971.7.1.3

[B116] Moon S.-K. Alaverdashvili M. Cross A. R. Whishaw I. Q. (2009). Both compensation and recovery of skilled reaching following small photothrombotic stroke to motor cortex in the rat. Exp. Neurol. 218, 145–153 10.1016/j.expneurol.2009.04.021 19409894

[B117] Navon D. (1977). Forest before trees: the precedence of global features in visual perception. Cogn. Psychol. 9, 353–383 10.1016/0010-0285(77)90012-3

[B118] Nelson C. A. (2001). The development and neural bases of face recognition. Infant Child Dev. 10, 3–18 10.1002/icd.239

[B119] Ogden J. A. (1993). Visual object agnosia, prosopagnosia, achromatopsia, loss of visual imagery, and autobiographical amnesia following recovery from cortical blindness: case M.H. Neuropsychologia 31, 571–589 10.1016/0028-3932(93)90053-3 8341415

[B120] Pascalis O. de Haan M. Nelson C. A. (2002). Is face processing species-specific during the first year of life? Science 296, 1321–1323 10.1126/science.1070223 12016317

[B121] Passarotti A. M. Paul B. M. Bussiere J. R. Buxton R. B. Wong E. C. Stiles J. (2003). The development of face and location processing: an fMRI study. Dev. Sci. 6, 100–117 10.1111/1467-7687.0025921128874

[B122] Paulus W. (2011). Transcranial electrical stimulation (tES—tDCS; tRNS, tACS) methods. Neuropsychol. Rehabil. 21, 602–617 10.1080/09602011.2011.557292 21819181

[B123] Pellicano E. Rhodes G. (2003). Holistic processing of faces in preschool children and adults. Psychol. Sci. 14, 618–622 10.1046/j.0956-7976.2003.psci_1474.x 14629695

[B124] Pellicano E. Rhodes G. Peters M. (2006). Are preschoolers sensitive to configural information in faces? Dev. Sci. 9, 270–277 10.1111/j.1467-7687.2006.00489.x 16669797

[B125] Pennington B. F. (2001). Genetic methods, in Handbook of Developmental Cognitive Neuroscience, eds Nelson C. A. Luciana M. (Cambridge, MA: MIT Press), 149–158

[B126] Peterson M. F. Eckstein M. P. (2012). Looking just below the eyes is optimal across face recognition tasks. Proc. Natl. Acad. Sci. U.S.A. 109, 19525–19526 10.1073/pnas.1214269109 23150543 PMC3511732

[B127] Polster M. R. Rapcsak S. Z. (1996). Representations in learning new faces: evidence from prosopagnosia. J. Int. Neuropsychol. Soc. 2, 240–248 10.1017/S1355617700001181 9375190

[B128] Powell J. Letson S. Davidoff J. Valentine T. Greenwood R. (2008). Enhancement of face recognition learning in patients with brain injury using three cognitive training procedures. Neuropsychol. Rehabil. 18, 182–203 10.1080/09602010701419485 18350413

[B129] Pozzulo J. D. Lindsay R. C. L. (1998). Identification accuracy of children vs. adults: a meta-analysis. Law Hum. Behav. 22, 549–570 10.1023/A:1025739514042 9833566

[B130] Rhodes G. Ewing L. Hayward W. G. Maurer D. Mondloch C. J. Tanaka J. W. (2009). Contact and other-race effects in configural and component processing of faces. Br. J. Psychol. 100, 717–728 10.1348/000712608X396503 19228441

[B131] Riddoch M. J. Humphreys G. W. (1994). Cognitive Neuropsychology and Cognitive Rehabilitation. London: Erlbaum

[B132] Rimmele U. Hediger K. Heinrichs M. Klaver P. (2009). Oxytocin makes a face in memory familiar. J. Neurosci. 29, 38–42 10.1523/JNEUROSCI.4260-08.2009 19129382 PMC6664913

[B133] Robbins R. McKone E. (2003). Can holistic processing be learned for inverted faces? Cognition 88, 79–107 10.1016/S0010-0277(03)00020-9 12711154

[B134] Robbins R. Nishimura M. Mondloch C. Lewis T. Maurer D. (2010). Deficits in sensitivity to spacing after early visual deprivation in humans: a comparison of human faces, monkey faces, and houses. Dev. Psychobiol. 52, 775–781 10.1002/dev.20473 20564328

[B135] Ross L. A. McCoy D. Wolk D. A. Coslett H. B. Olson I. R. (2010). Improved proper name recall by electrical stimulation of the anterior temporal lobes. Neuropsychologia 48, 3671–3674 10.1016/j.neuropsychologia.2010.07.024 20659489

[B136] Sangrigoli S. de Schonen S. (2004). Effect of visual experience on face processing: a developmental study of inversion and non-native effects. Dev. Sci. 7, 74–87 10.1111/j.1467-7687.2004.00324.x 15323120

[B137] Sangrigoli S. Pallier C. Argenti A.-M. Ventureyra V. A. G. de Schonen S. (2005). Reversibility of the other-race effect in face recognition during childhood. Psychol. Sci. 16, 440–444 10.1111/j.0956-7976.2005.01554.x 15943669

[B138] Savaskan E. Ehrhardt R. Schulz A. Walter M. Schachinger H. (2008). Post-learning intranasal oxytocin modulates human memory for facial identity. Psychoneuroendocrinology 33, 368–374 10.1016/j.psyneuen.2007.12.004 18221838

[B139] Schmalzl L. Palermo R. Green M. Brunsdon R. Coltheart M. (2008). Training of familiar face recognition and visual scan paths for faces in a child with congenital prosopagnosia. Cogn. Neuropsychol. 25, 704–729 10.1080/02643290802299350 18720102

[B140] Schultz R. T. (2005). Developmental deficits in social perception in autism: the role of the amygdala and fusiform face area. Int. J. Dev. Neurosci. 23, 125–141 10.1016/j.ijdevneu.2004.12.012 15749240

[B141] Schwarzer G. Huber S. Gruter M. Gruter T. Groß C. Hipfel M. (2007). Gaze behaviour in hereditary prosopagnosia. Psychol. Res. 71, 583–590 10.1007/s00426-006-0068-0 16767465

[B142] Sergent J. Villemure J.-G. (1989). Prosopagnosia in a right hemispherectomized patient. Brain 112, 975–995 10.1093/brain/112.4.975 2775997

[B143] Sitzer D. I. Twamley E. W. Jeste D. V. (2006). Cognitive training in Alzherimer's disease: a meta-analysis of the literature. Acta Psychiatr. Scand. 114, 75–90 10.1111/j.1600-0447.2006.00789.x 16836595

[B144] Snowball A. Tachtsidis I. Popescu T. Thompson J. Delazer M. Zamarian L. (2013). Long-term enhancement of brain function and cognition using cognitive training and brain stimulation. Curr. Biol. 23, 987–992 10.1016/j.cub.2013.04.045 23684971 PMC3675670

[B145] Sparr S. A. Jay M. Drislane F. W. Venna N. (1991). A historic case of visual agnosia revisited after 40 years. Brain 114, 789–800 10.1093/brain/114.2.789 2043949

[B146] Spillmann L. Laskowski W. Lange K. W. Kasper E. Schmidt D. (2000). Stroke-blind for colors, faces and locations: partial recovery after three years. Restor. Neurol. Neurosci. 17, 89–103 Available online at: http://iospress.metapress.com/content/u9g6b42x0l73v5vp/?genre=article&issn=0922-6028&volume=17&issue=2&spage=89 22387737

[B147] Strauss E. Wada J. Hunter M. (1992). Sex-related differences in the cognitive consequences of early left-hemisphere lesions. J. Clin. Exp. Neuropsychol. 14, 738–748 10.1080/01688639208402859 1474142

[B148] Sugimoto A. Koyama S. Midorikawa A. Futamura A. Ishiwata K. Ishii K. (2012). Is this a new type of primary prosopagnosia, both progressive and apperceptive? Neuropsychiatr. Dis. Treat. 8, 169–173 10.2147/NDT.S30541 22570548 PMC3346056

[B149] Susilo T. Duchaine B. (2013). Advances in developmental prosopagnosia research. Curr. Opin. Neurobiol. 23, 423–429 10.1016/j.conb.2012.12.011 23391526

[B150] Tanaka J. W. Lincoln S. Hegg L. (2003). A framework for the study and treatment of face processing deficits in autism, in The Development of Face Processing, eds Leider H. Schwarzer G. (Berlin: Hogrefe), 101–110

[B151] Tanaka J. W. Pierce J. (2009). The neural plasticity of other-race face recognition. Cogn. Affect. Behav. Neurosci. 9, 122–131 10.3758/CABN.9.1.122 19246333

[B152] Tanaka J. W. Wolf J. M. Klaiman C. Koenig K. Cockburn J. Herlihy L. (2010). Using computerized games to teach face recognition skills to children with Autism Spectrum Disorder: the *Let's Face It!* program. J. Child Psychol. Psychiatry 51, 944–952 10.1111/j.1469-7610.2010.02258.x 20646129

[B153] Taylor M. J. Batty M. Itier R. J. (2004). The faces of development: a review of early face processing over childhood. J. Cogn. Neurosci. 16, 1426–1442 10.1162/0898929042304732 15509388

[B154] Temple C. (1997). Developmental Cognitive Neuropsychology. Sussex: Psychology Press

[B155] Terney D. Chaieb L. Moliadze V. Antal A. Paulus W. (2008). Increasing human brain excitability by transcranial high-frequency random noise stimulation. J. Neurosci. 28, 14147–14155 10.1523/JNEUROSCI.4248-08.2008 19109497 PMC6671476

[B156] Thomas C. Avidan G. Humphreys K. Jung K. Gao F. Behrmann M. (2009). Reduced structural connectivity in ventral visual cortex in congenital prosopagnosia. Nat. Neurosci. 12, 29–31 10.1038/nn.2224 19029889 PMC5989137

[B157] Thomas M. Karmiloff-Smith A. (2003). Modelling language acquisition in atypical phenotypes. Psychol. Rev. 110, 647–682 10.1037/0033-295X.110.4.647 14599237

[B158] Thomas M. S. C. (2003). Limits on plasticity. J. Cogn. Dev. 4, 95–121 10.1080/15248372.2003.9669684

[B159] Valentine T. Powell J. Davidoff F. Letson S. Greenwood R. (2006). Prevalence and correlates of face recognition impairments after acquired brain injury. Neuropsychol. Rehabil. 16, 272–297 10.1080/09602010500176443 16835152

[B160] Wang P. P. Doherty S. Rourke S. B. Bellugi U. (1995). Unique profile of visuo-perceptual skills in a genetic syndrome. Brain Cogn. 29, 54–65 10.1006/brcg.1995.1267 8845123

[B161] Warrington E. K. James M. (1967). An experimental investigation of facial recognition in patients with unilateral cerebral lesions. Cortex 3, 317–326 10.1016/S0010-9452(67)80020-0

[B162] Wilkinson D. Ko P. Kilduff P. McGlinchey R. Milberg W. (2005). Improvement of a face perception deficit via subsensory galvanic vestibular stimulation. J. Int. Neuropsychol. Soc. 11, 925–929 10.1017/S1355617705051076 16519272

[B163] Wilson B. A. (1987). Rehabilitation of Memory. New York, NY: Guilford Publications

[B164] Wilson B. A. (2003). The theory and practice of neuropsychological rehabilitation: an overview, in Neuropsychological Rehabilitation: Theory and Practice, ed Wilson B. A. (Lisse: Swets and Zeitlinger), 1–10

[B165] Wilson B. A. Patterson K. (1990). Rehabilitation for cognitive impairment: does cognitive psychology apply? Appl. Cogn. Psychol. 4, 247–260 10.1002/acp.2350040403

[B166] Wilson C. E. Palermo R. L. Schmalzl L. Brock J. (2010). Specificity of impaired facial identity recognition in children with suspected developmental prosopagnosia. Cogn. Neuropsychol. 27, 30–45 10.1080/02643294.2010.490207 20623389

[B167] Wilson R. R. Blades M. Pascalis O. (2007). What do children look at in an adult face with which they are personally familiar? Br. J. Dev. Psychol. 25, 375–382 10.1348/026151006X159112

[B168] Yardley L. McDermott L. Pisarski S. Duchaine B. C. Nakayama K. (2008). Psychosocial consequences of developmental prosopagnosia: a problem of recognition. J. Psychosom. Res. 65, 445–451 10.1016/j.jpsychores.2008.03.013 18940375

[B169] Young A. W. Hay D. C. McWeeny K. H. Flude B. M. Ellis A. W. (1985). Matching familiar and unfamiliar faces on internal and external trials. Perception 14, 737–746 10.1068/p140737 3837875

[B170] Zihl J. von Cramon D. (1986). Zerebrale Sehstörungen. Stuttgart: Kohlhammer

